# Virtual Community Engagement Studio (V-CES): Engaging Mothers With Mental Health and Substance Use Conditions in Research

**DOI:** 10.3389/fpsyt.2022.805781

**Published:** 2022-06-15

**Authors:** Yaara Zisman-Ilani, Jennifer Buell, Shayna Mazel, Shannon Hennig, Joanne Nicholson

**Affiliations:** ^1^Department of Social and Behavioral Sciences, College of Public Health, Temple University, Philadelphia, PA, United States; ^2^Department of Clinical, Educational and Health Psychology, Division of Psychology and Language Sciences, University College London, London, United Kingdom; ^3^Institute for Behavioral Health Schneider Institutes for Health Policy, Heller School for Social Policy and Management, Brandeis University, Waltham, MA, United States; ^4^Maternal Mental Health Research Collaborative, Calgary, AB, Canada

**Keywords:** community engagement, co-production, parents with mental illness, mothers, mental health, substance use disorder

## Abstract

Active engagement of community stakeholders is increasingly encouraged in behavioral health research, often described as a co-production approach. Community stakeholders (e.g., patients, providers, policy makers, advocates) play a leading role together with research investigators in conducting the various phases of research, including conceptualization, design, implementation, and the interpretation and dissemination of findings. The concept of co-production has promising benefits for both the target population and the research outcomes, such as producing person-centered interventions with greater acceptability and usability potential. However, it is often the case that neither researchers nor community members are trained or skilled in co-production methods. The field of behavioral health research lacks tools and methods to guide and promote the engagement of diverse stakeholders in the research process. The purpose of this methods paper is to describe the Virtual Community Engagement Studio (V-CES) as a new method for engaging vulnerable populations like mothers with mental health and substance use conditions in research. We piloted the method in collaboration with the Maternal Mental Health Research Collaborative (MMHRC), focusing on one of the most vulnerable, under-researched populations, mothers coping with mental health and/or substance abuse disorders. Our pilot included mothers and providers who work with them as Community Experts to inform all phases of research design and implementation, and the interpretation and application of findings. The aim of this article is to describe the V-CES as a powerful tool that supports the engagement of mothers with mental health and/or substance use disorders and other community stakeholders in research, to provide examples of its use, and to make recommendations for future use, based on lessons learned. The V-CES toolkit is available for use with this target population as well as others.

## Introduction

The onset of COVID-19 and its emotional, social and psychological implications, particularly for mothers of childbearing age (e.g., social isolation, working at home with children, difficulty accessing treatment, loss of employment) have been found to be associated with a surge in use of substances (e.g., opioids, cannabis, alcohol), anxiety and depression (1, 2). Mothers of childbearing age with pre-existing conditions of mental illness are at a particular higher risk for developing a substance use comorbidity (3, 4). Unfortunately, this vulnerable group of women is often less engaged in mental health services and substance use treatment programs, and even less engaged in research projects that presumably target their needs and challenges (5, 6). The low rates of engagement in research among this group directly affects the quality of the services and care they receive, as new interventions and programs are being developed without the critical input of this vulnerable group of potential end-users or beneficiaries.

Co-production or co-design approaches are offered as processes for tailoring interventions and treatment programs, making them more relevant to the lived experience of the target population and, as a result, potentially increasing service/treatment engagement and effectiveness (7, 8). In a co-production process, community stakeholders (e.g., patients, providers, policy makers, advocates) and research investigators partner in conducting the various phases of research, including conceptualization, design, implementation, and dissemination. A co-production approach can potentially lead to more meaningful and impactful programs, interventions and outcomes for both patients and researchers (9). Co-production is also associated with benefits to researchers, such as enhanced acceptability and feasibility of methods and procedures, enhanced relevance of outcomes in terms of meaning and impact for patients, and enhanced sustainability of interventions (10, 11). Similar to other engagement approaches, such as shared decision making (12, 13), benefits for patients/participants in co-production include improved quality of care and outcomes, and feeling valued and empowered by sharing their experiences and expertise on behalf of “a greater good” – that is, informing research and practice approaches that may improve the lives of others. Patients/community stakeholders may also benefit from the sense of mastery that emerges from being “in the driver's seat,” not just as passive patients or research subjects, but as significant decision-makers (10).

Despite the promising potential, the use and implementation of co-production in practice is limited (14). Researchers may be challenged in engaging community members (e.g., patients, providers, policy makers, and advocates) as partners in research endeavors rather than, or in addition to, as participants (15). Many researchers are not trained or skilled in identifying, recruiting, convening and engaging community stakeholders or preparing them for participation in research in an advisory capacity or as contributing members of a research team. At the same time, mothers with mental or substance use disorders may not feel comfortable participating, may be distrusting of researchers, or may have concerns that there could be possible legal or social services consequences to their involvement, due to stigma and the sensitive nature of maternal mental illness and substance use (16).

The COVID-19 pandemic has given rise to the rapid digitalization of remote mental health via telemedicine and digital psychiatry approaches (17, 18). Attention is also being given to the development of virtual research methods, with the Patient-Centered Outcomes Research Institute (PCORI) in the United States soliciting projects via its Eugene Washington Engagement Award program to develop and enhance community engaged research approaches in the virtual era imposed by COVID-19 pandemic. This article describes the results of a PCORI engagement award to develop the Virtual Community Engagement Studio (V-CES) method for virtually engaging this vulnerable target population, mothers of childbearing age with mental health and/or substance use disorder, across the research life cycle. We will describe the V-CES method and its application including feedback from participants, lessons learned, and recommendations.

## Materials and Equipment

### V-CES Background

The V-CES was developed based on the community engagement studio (CES) model (19–21) as an interactive method to facilitate co-production in behavioral health research with vulnerable populations, in our case, mothers with mental health and/or substance use disorders. The end goal was to engage community stakeholders as experts with researchers in various steps of behavioral health research to provide input to inform and improve recruitment procedures, data collection, ethical considerations, the choice of outcomes, and the interpretation and dissemination of findings. The V-CES is inspired by the CES approach that focuses on supporting the involvement of community members with researchers to inform next steps in research and treatment innovation (19, 20). The original CES was developed by Joosten and colleagues at Vanderbilt University to recruit and train stakeholders and prepare researchers to participate in an in-person meeting (19). The V-CES and the original in-person CES model describe research efforts with people rather than on people, similar to existing community participatory based research (CBPR) or Cooperative Inquiry (CI) approaches. Since we adapted the CES method during and in response to the COVID-19 pandemic, the community engagement studio takes place in a virtual/digital medium (i.e., an online video conference space), which requires consideration of the differences from engaging with community stakeholders in-person. Conducting the CES virtually provides opportunities to facilitate and broaden the engagement of diverse mothers (e.g., race, ethnicity, disability) in diverse geographic areas (e.g., rural, urban), time zones, and living situations (e.g., alone or with extended family), with diverse responsibilities (e.g., caring for young children at home) that impact on scheduling (e.g., during school hours or after school) with researchers from different institutes or universities, states or countries, and areas of research.

### V-CES Structure

The V-CES method includes two components: The core V-CES team, which is at the center of the V-CES activity, and the operational team, working behind the scenes, that administratively supports the operation and execution of the V-CES. Importantly, both components reflect a commitment to a partnership between the researcher and the target population (i.e., mothers with mental and/or substance use disorder). The core V-CES team is composed of three types of members: researchers, Community Experts, and Facilitators. The V-CES is implemented by the operational team that includes a Community Navigator, Science Navigator, and a Manager. The operational team is responsible for implementing the V-CES, provides coaching and support to both researchers and Community Experts, and manages administrative aspects such as logistics and resource preparation, including video conference platform, V-CES recruitment, and the solicitation of feedback from V-CES participants.

#### The V-CES Core Team

##### Researchers

The researcher identifies a theme or topic area and prepares a short presentation to discuss with the V-CES team in preparation for the V-CES. The V-CES team recommends changes to the presentation as needed to improve clarity and ensure the language and tone are appropriate and sensitive to the Community Experts' characteristics and experiences. Researchers are guided to avoid jargon, technical terms and acronyms and encouraged to use plain language. The researcher's opening V-CES presentation serves to elicit feedback from the Community Experts on how best to move forward with a research project. After making the presentation, the researcher's role is primarily to listen, asking and answering questions for clarification. Participants may want to know, for example, why a researcher is interested in a particular topic.

##### Community Experts

Community members, be they mothers, family members, peer specialists, service providers or advocates, are considered experts by experience and are the key to the success of the V-CES. Ideally, they represent diverse backgrounds and are connected to the community in various ways. For example, a care manager or peer specialist who works with mothers in metal health/substance use treatment may have a very different, but equally valuable, perspective from a mother currently in recovery with a similar condition. Generally, Community Experts should have good verbal communication and listening skills, a desire to learn about research, and a willingness to share their experiences. Accommodations can be made to support the engagement of participants whose skills may be compromised by a health condition or disability.

##### Facilitator

As recommended by Joosten et al. (19) the Facilitator's task is to create a comfortable, safe environment that allows for open and frank discussion and to guide the conversation among researchers and Community Experts. A skilled Facilitator does not interject their opinions or biases into the conversation. The Facilitator should have professional and/or lived experience working within the target population community and possess the ability to balance the differences in power that can naturally occur when researchers and community members come together. The Facilitator's responsibilities include explaining (and keeping) discussion ground rules (e.g., be concise, don't interrupt, and maintain confidentiality), keeping the discussion on track, using the predefined questions as the discussion framework, and guiding the discussion, only interjecting their own opinion and personal observations with intention and purpose.

#### The V-CES Operational Team

##### Community Navigator

A boundary spanner with familiarity with the target community, experience with academic-community partnerships, and understanding of principles of community engagement is a good candidate for the Community Navigator role. The navigator should have experience building rapport and trusting relationships with key community leaders. Specific responsibilities include helping to identify, orient, and support Community Experts who participate in the V-CES; coaching the researchers on communicating with Community Experts with personal or professional experience with maternal mental health and/or substance use; and developing and maintaining mechanisms to communicate with community partners, increase interaction between community partners and researchers, and track the development of research-community partnerships. Hiring a Community Navigator from the community puts into practice fundamental principles of community engagement such as mutual benefit, respect and community capacity building (19). A respected community member is likely to have access to networks unfamiliar to someone who works in an academic/research setting.

##### Science Navigator

The Science Navigator provides guidance on identifying and recruiting participating researchers, and coaches them on communicating effectively with non-researchers and engaging Community Experts as consultants, rather than as research subjects (19). The Science Navigator benefits from having experience in patient-centered outcomes research, community-engagement, comparative effectiveness and community-based participatory research (19). Specific responsibilities include helping to identify, orient, and support researchers who participate in the V-CES; coaching the researchers on communicating effectively with non-researchers and on engaging Community Experts as consultants; and encouraging Researchers to consult Community Experts who would like to remain involved as the research project develops.

##### Manager

The Manager works with the Community Navigator and Science Navigator to reach out to selected researchers and Community Experts, securing the time and access code to virtual sessions (e.g., via ZOOM, SKYPE, TEAMS) for the V-CES, and preparing necessary materials to assist, plan, and implement the V-CES. Specific responsibilities include managing logistics such as securing a virtual space and time for the V-CES that are convenient for the Community Experts; making sure the appropriate documentation is completed for each V-CES, including capturing the Community Expert feedback from each session; and the completion of evaluation surveys and forms needed to process payments or stipends for participation.

## Methods

Step by step procedures for implementing a V-CES are described below, using examples from the application of the V-CES method with researchers and the target population of mothers with mental and/or substance use disorders and providers who work with them in the community. We implemented four V-CES's during the COVID-19 pandemic with a total of 19 Community Experts (i.e., mothers and providers). The first two sessions were conducted in April 2020 and included 16 participants: researcher, facilitator, manager and 13 Community Expert, White women, ages 25 to 45, from four states in the US. A third V-CES was held in March 2021 and included 6 participants: researcher, facilitator, manager and 3 Community Experts, White women from the Massachusetts area. A fourth V-CES was conducted in May 2021 with 8 participants: researcher, facilitator, manager and 5 Community Experts, White women, one identifying as Hispanic/Latina, ages 25 to 55 from six states in the US. The V-CES procedures were reviewed by the Brandeis University Institutional Review Board and deemed to be exempt from consideration as Human Subjects Research. Community Experts received gift cards in the amount of $150 US.

### Recruitment

#### Recruitment of Researchers

The Operational Team was responsible for the recruitment process. The V-CES Manager centralized the recruiting process with the help of the Community and Science Navigators. We used two strategies to recruit researchers and Community Experts for the V-CES sessions. Researchers were recruited by the Science Navigator from a mapped pool of researchers who focus on maternal mental health and/or opioid use/recovery. Interested researchers were invited to submit a paragraph describing their research and plans for community engagement including a summary of the problems or questions their project would address, target population, stage of research, and questions they wanted to propose to Community Experts along with feedback needed. The V-CES team chose four research projects problems or questions that were most likely to benefit from input from or be of interest to available Community Experts.

#### Recruitment of Community Experts

To recruit mothers with mental and/or substance use disorders as Community Experts, we reached out through the Maternal Mental Health Research Collaborative (MMHRC) listserv and social media platforms. First, we emailed a survey to the MMHRC listserv. The survey was designed to query mothers who would be interested in participating as Community Experts regarding contact information and availability as well as basic personal and/or professional mental health and/or substance use experiences to help build an appropriate Community Expert pool. We published the survey link online to reach potential participants who were members of the MMHRC Facebook group, a social media initiative for reaching mothers coping with maternal mental health conditions and/or substance abuse. The Manager or the Community Navigator contacted potentially interested Community Experts to set up an additional screening conversation via telephone or online platform. The purpose of this additional screening step was to confirm potential Community Experts' interest, availability, and comfort level participating in an online group discussion on challenges facing mothers and how to improve research on maternal mental health and/or substance use. An important part of the screening process was also ensuring that Community Experts have a relatively quiet and private place where they feel comfortable talking about sensitive topics, and the technology tools and skills to participate virtually.

### Preparation of Participants

Preparing Community Experts and researchers for the V-CES and buttressing their sense of agency is an important step in the V-CES process. Prior to the V-CES, the team emailed the V-CES participants a guide that provided a general description of process and the role of the Community Experts and researchers; and an online survey that captured general background information. Participants were asked to review and complete these materials prior to the V-CES. Participants were encouraged to contact the Operational Team with any comments and/or questions. We prepared a series of videos of researchers addressing questions provided by mothers regarding research – Research 101 for Mothers (https://research4moms.com/research-101/). Mothers provided video clips for researchers regarding research participation (https://research4moms.com/research-101/). These videos were available to participants who requested further information about the project.

#### The V-CES Process

In the introduction of a V-CES meeting, Community Experts and the researcher were informed by the Facilitator that the Science Navigator would take notes. Participants were encouraged to have their video cameras on, if they were comfortable. The V-CES Manager and/or the Facilitator asked for participants' permission to record the meeting. This recording was only used as a reference for notes and would never be shared without permission from participants. The steps in conducting the V-CES included: greetings and introductions, providing a brief overview of the purpose of the meeting and the process of discussion and communication (“discussion roles”), and the researcher's presentation. The Facilitator kept the conversation on track, making sure everyone's voice was heard and the two to three research topics were addressed. The Science Navigator took notes throughout the discussion and, finally, the Community Navigator thanked everyone for their participation and explained next steps.

#### V-CES Follow-Up

The V-CES follow up materials provided the researcher important feedback from the Community Experts and facilitated further collaboration between the researcher and the Community Experts.

##### Follow-Up for the Researcher

A summary report including the Science Navigator's notes and verbatim written comments from the Community Experts was shared with the researcher within one week of the V-CES. We highlighted specific recommendations as related to the topics that were discussed during the meeting (21). The researcher also received a one-page Continuing Community Engagement Guide that suggested ways for the researcher to maintain appropriate communication with the Community Experts who reported interest in serving as consultants as the research project develop.

##### Follow-Up for the Community Experts

We notified participants of any changes, adjustments and improvements to the research made as a result of their input. Items shared as follow-up could include updated outreach materials, policy and procedural changes or significant accomplishments of the study due to advice received during the V-CES. If possible, it would be important to provide Community Experts periodic updates on the project as well as any findings published or disseminated by the researcher (21). Community Experts participating in the V-CES sessions contributed to and had opportunity to review tip sheets for researchers and mothers developed as part of the project (See Supplementary Material). The V-CES toolkit is also provided as Supplementary Material.

## Results

### Recruitment via Social Media

Our recruitment advertisement via the MMHRC listserv and Facebook group resulted in 91 mothers contacting us. We were able to strategically select an average of 5 mothers for each of the V-CES sessions and followed specific email invitations with a screening call by the V-CES Manager. We recruited more participants than needed in case a potential participant had a last-minute schedule conflict or childcare challenge.

### Community Experts' Feedback

Community Experts who participated in the V-CES reported positive experiences. They were grateful for the opportunity to impact research and wanted more opportunities to participate in such co-production initiatives in the future. For example, one participant shared she felt “*…being heard and listened to [by the researcher]. I appreciate the time that was put into this”* (V-CES March 21). Another participant shared that she “*…like the fact that the researcher is trying to reach out to women during the first year after giving birth. I know it's a difficult time for many women, but so critical to understand for others”* (V-CES May 21).

Community Experts felt empowered by their research co-production experience and wanted to continue their involvement in the future to impact and disseminate research on maternal mental health and substance use issues:

“*I loved that this researcher really wanted to figure out how we can add supports and not let these moms slip through the cracks. The researcher is motivated by identifying this vulnerable population and providing them with support”* (V-CES May 21)

“*I liked the connections…I liked the diversity of professionals…I liked the empathy…I liked the desire to improve services, I liked that [the researcher] wanted to know what we think is working and not working and where we would like to see services go in the future.”* (V-CES May 21)

“*It was so helpful having all 3 experts from different geographic locations and within different medical communities - rural, large city, and small city; but we all had similar experiences. This says so much about how change needs to be widespread and proves to be a big challenge*.” (V-CES March 21)

Participation in research co-production also has the potential to impact participants' recovery:

“*Getting new mothers to talk about their substance use to begin with. It's a huge step for these moms to come forth to talk about addiction, or even admit they are experiencing addiction. It's scary and full of judgement from others. The stigma around addiction and mental health needs to be educated with the entire community. Hopefully moms will see the benefit of this program and trust the process” (*V-CES May 2021)

Last, Community Experts provided essential feedback for research conducted with mothers with mental health and substance use:

“*I think that the idea of a flyer seems very non-threatening along with all the other paperwork that gets sent home with a new mom when she's discharged from the hospital. I had an entire folder. As we talked about the questions, it was really important to make the surveys more conversational in tone.”* (V-CES May 21)

“*I would say disclosure about substance use or any mental health that the participant maybe going through [is an issue]. As a mom, they [research participants] may feel judged or would be scared that something may happen to their baby if anyone knows what they are going through. Another challenge may be retention, having a person fill out a survey every month could be challenging, but I think with good incentives, it may make it a little easier.”* (V-CES May 21)

“*It's a huge step for these moms to come forth to talk about addiction, or even admit they are experiencing addiction. It's scary and full of judgement from others. The stigma around addiction and mental health needs to be educated with the entire community. Hopefully moms will see the benefit of this program and trust the process.”* (V-CES May 21)

### Researchers' Feedback

We learned that even when working with researchers who have previous community engagement experience, it was important and necessary to coach researchers on how to engage with Community Experts effectively. We found that for a successful and collaborative conversation Community Experts want: 1). to understand the researcher's motivation, so researchers should be willing and able to talk about their commitment to the topic, professionally and perhaps, personally; 2). to know how their input will specifically impact the research project and then the broader community; and 3). to feel heard as knowledgeable consultants. We had researchers create brief video overviews to introduce themselves and their interests, provided to participants prior to the event. The team reviewed their brief presentations in advance of the V-CES and provided feedback. Researchers were challenged by having a conversation in plain language with mothers and stakeholders whose perspectives they were hoping to solicit. They benefitted from coaching prior to V-CES sessions, as well as guidance and direction during sessions.

researchers reported that their perception about the role of patient or community stakeholders in their research changed as a result of the V-CES:

“*I'm thinking about their interactions with the medical community and stigmatization and 'othering' they talked about*.” (V-CES April 2020).

“*They [Community Experts] had a fantastic understanding of the recruitment process.”* (V-CES May 2021).

## Discussion

This article describes the efforts and steps taken to develop and implement V-CES, a co-production engagement method to involve patients and community stakeholders in the design and implementation of research projects, and the interpretation and dissemination of findings. The V-CES method is based on the CES model and was developed in response to the barriers and challenges in community engaged research caused by the breakout of the COVID-19 pandemic. Our experience demonstrates the potential contribution to V-CES to improve research engagement and relevancy in the virtual space post-pandemic, allowing for increased diversity of participants, communities, and service contexts. For researchers and Community Experts, in our case mothers with mental health and substance use, V-CES participation provided a “win-win” scenario, with the potential to improve recruitment efforts and make research outcomes more personalized, meaningful, and relevant.

Our specific V-CES pilot had three main limitations. First, by nature, the V-CES excludes populations without access to the internet or those who are not comfortable using online platforms. Therefore, participants were those with stable internet access, who felt comfortable using a virtual platform (Zoom) and were able to be in a safe, convenient location during their participation in a V-CES. As with other virtual remote approaches, it is important to recognize that the V-CES may be less accessible or effective for individuals who have no or limited access to the internet and those who prefer in-person interaction for many reasons (22). One benefit of the V-CES, compared to other digital-virtual approaches, is the existence of an in-person model, the original CES, that allows for the inclusion of populations who do not have or may benefit less from a virtual model.

Second, most Community Experts were White from Northeastern and Midwestern states. Recruiting a racially diverse group was difficult because a primary source for recruitment was the MMHRC Facebook group page, where most members are Northeastern/Midwestern US white women (3). Future use of the V-CES method should purposefully address diverse Community Expert populations. Last, due to the nature of the study (co-production participatory design), we did not collect information about what kind of mental health issues and/or SUDs recruited mothers experience, which may limit the replicability of the method in different subpopulations.

To summarize, the V-CES is a potentially useful approach for operationalizing co-production processes virtually, which is beneficial during emergencies (i.e., COVID-19) (23) but also for those living in rural areas, lacking transportation, or balancing work schedules and responsibilities at home, or for those who are experiencing barriers to “classical participation”(24). While the V-CES model may well be useful to researchers and Community Experts implementing co-produced research in other health domains, future studies are required to contribute to the growing literature on the science of engagement. For example, our team has been awarded a PCORI engagement award to implement and further evaluate the V-CES with mothers with intellectual and developmental disabilities and behavioral health conditions, with community stakeholders, and researchers. The V-CES toolkit and tip sheets are available as Supplementary Material and on the MMHRC website (https://heller.brandeis.edu/ibh/affiliates/mmhrc/about.html). We welcome its further use, implementation, and evaluation by community stakeholders and researchers, and look forward to receiving feedback for further improvements and future studies.

## Data Availability Statement

The datasets presented in this article are not readily available to protect the participants' privacy. Requests to access the datasets should be directed to the corresponding author.

## Ethics Statement

The studies involving human participants were reviewed and approved by the V-CES procedures were reviewed by the Brandeis University Institutional Review Board and deemed to be exempt from consideration as Human Subjects Research. Written informed consent for participation was not required for this study in accordance with the national legislation and the institutional requirements.

## Author Contributions

All authors listed have made a substantial, direct, and intellectual contribution to the work and approved it for publication.

## Funding

Research reported in this article was funded through two Patient-Centered Outcomes Research Institute^®^ (PCORI^®^). Awards: #EAIN-00147 and #8285-BU. The views and statements presented in this article are solely the responsibility of the authors and do not necessarily represent the views of the Patient-Centered Outcomes Research Institute^®^ (PCORI^®^), its Board of Governors or Methodology Committee.

## Conflict of Interest

The authors declare that the research was conducted in the absence of any commercial or financial relationships that could be construed as a potential conflict of interest.

## Publisher's Note

All claims expressed in this article are solely those of the authors and do not necessarily represent those of their affiliated organizations, or those of the publisher, the editors and the reviewers. Any product that may be evaluated in this article, or claim that may be made by its manufacturer, is not guaranteed or endorsed by the publisher.
