# Supplementary material for: Virtual Community Engagement Studio (V-CES): Engaging Mothers With Mental Health and Substance Use Conditions in Research
Source: Front Psychiatry. 2022 Jun 15;13:805781. doi: 10.3389/fpsyt.2022.805781 (PMC9240264; doi:10.3389/fpsyt.2022.805781)
Supplement: Supplementary file 1 [file Data_Sheet_1.PDF]

Virtual Community Engagement Studio Toolkit:

For Researchers and Community Experts Interested in Maternal Mental Health and/or Substance Use/Recovery

The Maternal Mental Health Research Collaborative

Jennifer Buell, MPP
Shayna Mazel, MPP
Yaara Zisman-Ilani, PhD
Shannon Hennig, MA
Joanne Nicholson, PhD

June 2021

This toolkit was funded through a Patient-Centered Outcomes Research Institute (PCORI) Eugene Washington PCORI Engagement Award (EAIN-00147).
MMHRC © 2021

For more information or to download this toolkit see https:
https://heller.brandeis.edu/ibh/affiliates/mmhrc/index.html or research4moms.com

Brandeis | THE HELLER SCHOOL FOR SOCIAL POLICY AND MANAGEMENT
Institute for Behavioral Health

MMHRC
Maternal Mental Health Research Collaborative

Acknowledgments

This toolkit builds on the Community Engagement Studio (CES) model and Toolkit developed by the Meharry-Vanderbilt Community Engaged Research Core (CERC)[1]. The Maternal Mental Health Research Collaborative (MMHRC) adapted the CES model for Researchers and Community Experts interested in maternal mental health and/or opioid use/recovery to provide focused guidance for engaging these populations in research in the virtual medium. Our goal is to build on and add to the work done by the Meharry-Vanderbilt group, acknowledging their original contribution while adding the nuances appropriate to our target population and considerations relevant to virtual engagement. Some paragraphs within each numbered section have been removed or condensed, while original language may appear in other sections with appropriate acknowledgement.

The MMHRC is a peer-led, patient-centered initiative with research partners at the Heller School for Social Policy and Management at Brandeis University and Temple University's College of Public Health. MMHRC seeks to engage mothers with experience with maternal mental illnesses and/or substance use in research and in using research findings to inform health decision-making. The MMHRC is driven by a collective vision of developing a peer-led, patient-centered think-tank and research hub where mothers, healthcare providers, researchers and other stakeholders co-create research questions and strategies to address them. These goals are guided by a shared desire to quickly improve health by focusing on patient identified priorities. Partnership and collaboration with stakeholders are key tools employed by the MMHRC team.

PCORI is an independent, non-profit organization authorized by Congress in 2010. Its mission is to fund research that will provide patients, their caregivers, and clinicians with the evidence-based information needed to make better-informed healthcare decisions. PCORI is committed to continually seeking input from a broad range of stakeholders to guide its work. The statements presented in this toolkit are solely the responsibility of the author(s) and do not necessarily represent the views of the Patient-Centered Outcomes Research Institute (PCORI), its Board of Governors or Methodology Committee.

MMHRC is grateful to our community collaborators at the Institute for Health & Recovery, and the Massachusetts Department of Public Health, and the many Community Experts who helped us refine and tailor the CES model and toolkit for maternal mental health and/or opioid/substance use recovery research using a virtual medium.

[1] Israel et al. 2019

Table of Contents

1) Overview of a Virtual Community Engagement Studio (V-CES) 4

2) The Virtual Community Engagement Studio Team 13

3) The Facilitator 15

4) The Researcher 17

5) The Community Experts 19

6) Planning and Logistics 24

7) Follow-up 28

Appendix A: Facilitator V-CES Guide 32

Appendix B: Tools for Recruiting Researchers 37

Appendix C: Researcher V-CES Guide 40

Appendix D: Tools for Recruiting Community Experts 48

Appendix E: Community Expert V-CES Guide 52

Appendix F: Community Expert Initial Background Online Survey 57

Appendix G: Participant V-CES Forms and Surveys 60

Appendix H: Continuing Community Engagement Tips for Researchers 68

1) Overview of a Virtual Community Engagement Studio (V-CES)

How community stakeholders can impact research:

- Prioritize research topics
- Increase relevance to patients and communities
- Strengthen recruitment and retention efforts
- Help frame protocols and procedures
- Participate in data collection and analysis
- Assist with data interpretation
- Increase dissemination and uptake of research results

Engagement of community stakeholders in research can improve the quality and relevance of the research and outcomes.[2] Journals and funders increasingly require investigators to provide evidence of stakeholder involvement in framing and conducting research and disseminating research findings. Researchers in maternal mental health and/or substance use fields can particularly benefit from engaging with community members, whose perspectives may be invaluable to a person-centered approach for intervention development and testing, and for the uptake of evidence-based practices. Benefits of community engagement in research may be conveyed back to community participants, who feel valued and empowered by sharing their experiences and expertise on behalf of "a greater good" – informing research and practice approaches that may improve the lives of others.

It may be challenging for researchers to engage stakeholders and community members, such as patients, caregivers, health providers, advocates, and policy makers as partners in research endeavors, rather than as "subjects." Many researchers are not prepared to identify, recruit, convene and engage community stakeholders or prepare them for participation in research in an advisory capacity or as an active part of a research team. At the same time, community members may not feel comfortable participating, and may be distrusting of researchers, or may have concerns that there

[2] Mitchener et al. 2012; Green 2003; Johnston et al. 2015

could be possible legal or social services consequences to their involvement, due to stigma and the sensitive nature of maternal mental health and substance use.

The Virtual Community Engagement Studio (V-CES) provides researchers and stakeholders an interactive, efficient, and effective way to engage and provide meaningful input into all phases of research.
The V-CES model provides a framework for engaging community members in a way that is culturally sensitive and in keeping with their priorities, values and needs. Community engagement studios (CESs) provide community members the opportunity to inform next steps in research and treatment innovation[3]. A traditional CES takes place in person, while a virtual CES takes place using a virtual/digital medium. Conducting a CES virtually increases access to the CES for community members who may live in remote locations, can bring together stakeholders from different regions, provides opportunity for strategically engaging diverse participants, and is an efficient and cost-effective way to conduct a CES.

The Virtual Community Engagement Studio (V-CES) provides researchers and stakeholders an interactive, efficient, and effective way to engage and provide meaningful input into all phases of research.

In a V-CES, the **Researcher** gives a presentation about their project and poses specific questions to a group of **Community Experts**[4] (e.g., stakeholders including individuals with personal experience, family members, practitioners, advocates, and policy makers) in the virtual space, rather than in person. The Researcher uses language that is appropriate and sensitive to the Community Experts' experience, background and role, and poses and addresses questions of interest to the Community Experts. The discussion that follows is guided by a **Facilitator** to elicit authentic and constructive input and feedback on setting research priorities, developing and implementing a study, or disseminating research findings.

We have had experience in our V-CESs with: 1) developing a survey on the experiences of stigma among pregnant women who use opioids; 2) developing questions for upcoming research on how the COVID-19 pandemic changed support for women in recovery; 3) prioritizing research topics around how mothers experience healthcare and accessing mental health and substance use recovery services; and 4) designing recruitment strategies for postpartum mothers with histories of substance use disorders.

[3] Israel et al. 2019
[4] Israel et al. 2019

The V-CES is an **interactive, consultative model**; it is not a standing advisory board, a tool for recruiting research participants, or a data collection or research method such as a focus group. **The Community Experts who participate in the V-CES are not research subjects; rather, they are advisors and/or consultants** who receive a stipend for helping with this effort.

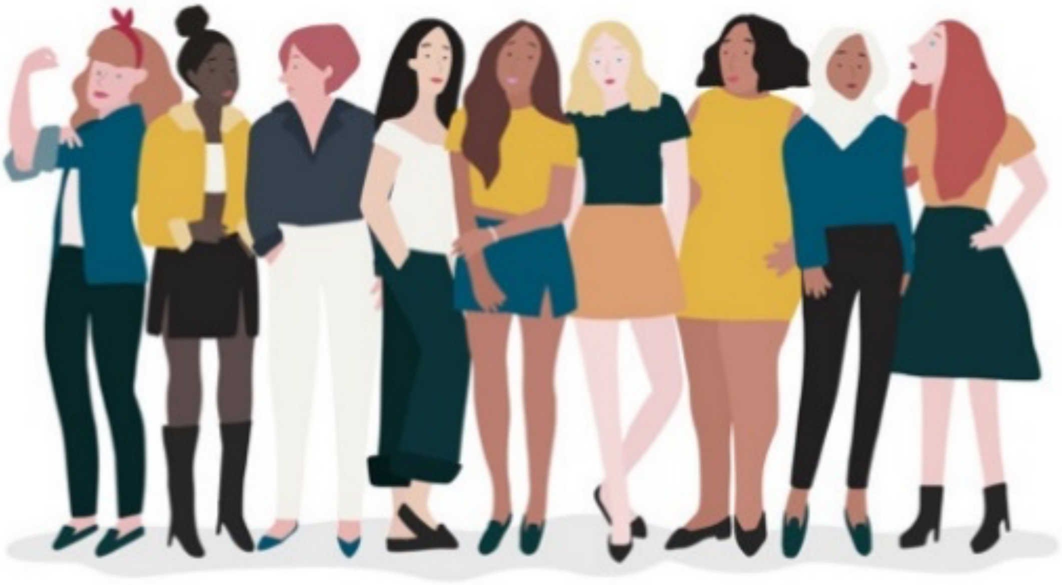

What is the Difference Between a Virtual Community Engagement Studio and a Focus Group?[5]

Components	Virtual Community Engagement Studio	Focus Group
**Purpose**	• Inform development, implementation or dissemination of research.	• Qualitative data collection.
**Approach**	• Multi-directional discussion	• Uni-directional
**Participants**	• Consultants.<br>• Identified as experts based on their personal and/or professional experience.	• Research subjects.<br>• Screened, informed consent obtained.
**Facilitator**	• Neutral – could be a community member.<br>• Not affiliated with a research project.<br>• Uses techniques to balance power; understands power dynamic with mothers with experiences with mental health issues and/or substance use, and is sensitive to their (possible) histories of trauma and other concerns.<br>• Uses guide for conversation: can diverge if relevant or appropriate.	• Research team member.<br>• Uses pre-approved script; cannot diverge.
**Preparation**	• Coaching for Researcher on managing potential power dynamics and communicating in plain language.<br>• Screening and guide for Community Experts regarding research, roles and expectations, and the value of lived experience.<br>• Background materials for Facilitator regarding research questions, participants' experiences, and Researcher's goals for the session.<br>• IRB requirements may depend on institutional review.	• IRB approval.<br>• Consenting of research subjects.
**Compensation**	• Consulting stipend.	• Participant stipend.

[5] Israel et al. 2019

**Use of Input**	• Participant comments and recommendations summarized.<br>• V-CES Team may help Researchers interpret and apply recommendations.<br>• Community Experts may be asked about their ability and willingness to participate in various stages of the research discussed.	• Participant comments transcribed.<br>• Transcription qualitatively analyzed.<br>• Findings reported in scientific journals and presentations.
**Follow-Up**	• Researchers are encouraged to continue to consult with Community Experts interested in remaining involved.	• None.

Partnering with Mothers and Others as Community Experts

Community Experts may be mothers or mothers-to-be with past or current experience of mental health conditions and/or substance use, family members, practitioners, advocates, or policy makers. Mothers at high risk often feel judged, and mothers with experiences of mental health conditions or substance use may feel even more vulnerable. They may have experienced negative attitudes and actions of others, who make assumptions about them, perhaps informed by damaging narratives and stereotypes. Mothers may have past experiences contributing to trauma, either themselves or as the witness of bad things happening to others.

Consequently, addressing issues of participants' comfort and safety are paramount in any engagement scenario. The most important rule of thumb is dependability – that is, doing what you say you will do, showing up, and following up. Community Experts should be provided with information ahead of time and throughout the V-CES, and given choices regarding their participation and the extent to which they may choose to draw from their lived experiences to inform the discussion. Participants who may have histories of victimization or violence may feel uncomfortable or find themselves responding in negative ways to characteristics of the V-CES or to information shared by others. Literature regarding this topic shows that participants who have experienced trauma regard the experience of sharing their stories for research positively even if it may be triggering.[6] However, it is important to be alert to this possibility and take care to guide the discussion in a sensitive and respectful manner.

Many women with mental health and substance use challenges may have had prior experiences with professionals or others who made negative assumptions about their capacity to parent their children safely.

It is also possible that some Community Experts, especially those who have experienced traumatic events, may feel uncomfortable with using a video function that "exposes" or "intrudes" into their home environment. In initial conversations with the Community Experts, the V-CES team should explain that participants can opt-out of the video function at any point during the V-CES.

Confidentiality and Consent

[6] Abu-Rus et al., 2019

The V-CES as described in this document was deemed to be exempt from review by the Institutional Review Board. Therefore, signed informed consent was not a part of our V-CES process. It is advisable to check with your organization or university's Institutional Review Board for their review and determination, and follow their procedures, as recommended.

Similar to a research study, however, Community Experts receive complete information and training on the V-CES process prior to and again at the beginning of the planned session and can opt-out of participation at any time along the way. As in any group situation (that may remind participants of group therapy, for example), it is advisable to remind participants that disclosure of any information is voluntary and to ask participants to respect the contributions of others. While the goal of the V-CES is to contribute to research, participants may choose and find it beneficial to share their experiences and explore common concerns, as these may well shape the research agenda or inform research procedures.

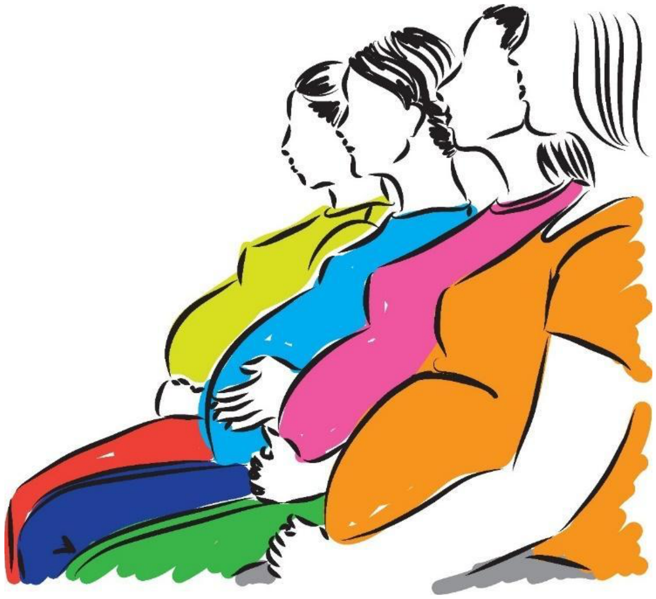

With this in mind, Facilitators or Researchers should refrain from asking Community Experts detailed or direct questions about their personal mental health or substance use status, beyond confirming their experience, as appropriate, for participation in the V-CES. It is important for participants to be mindful of this before engaging in the V-CES, and to be thoughtful and respectful during all encounters.

Helping Participants Feel Comfortable and Safe

Pregnant and parenting women with mental health and/or substance use issues may be, and may feel, particularly vulnerable to the judgments of others. Unfortunately, many women with mental health and substance use challenges may have had prior experiences with professionals or others who made negative assumptions about their capacity to parent their children safely. Admittedly, some of the concerns of others, in some circumstances, may have been justified. Mandatory reporting of the risk or suspicion of harm to self or others refers to the obligation or requirement for professionals who may have contact with vulnerable people (e.g., children, elders,

disabled individuals) to report suspected or observed abuse. Health services researchers who are licensed clinicians or service providers may be required by law or their code of ethics to take action if the likelihood of harm to self or others becomes apparent. Laws vary from state to state. People who are required to report abuse may include teachers, social workers, physicians, or probation officers, for example. A V-CES may include participants in different states, so it is important to be mindful of how laws differ among states. If you, as the Facilitator or Researcher, are in this position (e.g., a licensed mental health professional) and have any concerns about this issue, please check with your institutional review board or state licensing board for clarification of your responsibilities. Your Institutional Review Board may provide guidance.

If you decide that mandatory reporting is your obligation in a V-CES-type of scenario, it is important for you to acknowledge your responsibility at the beginning of the session and proceed with transparency. It has never happened, in our several decades of research with this patient population, that we, as researchers, have been obligated to file a report of abuse or neglect. However, it is your responsibility to clarify your role and responsibilities, and to be transparent about these issues in setting up and conducting a V-CES with any patient population.

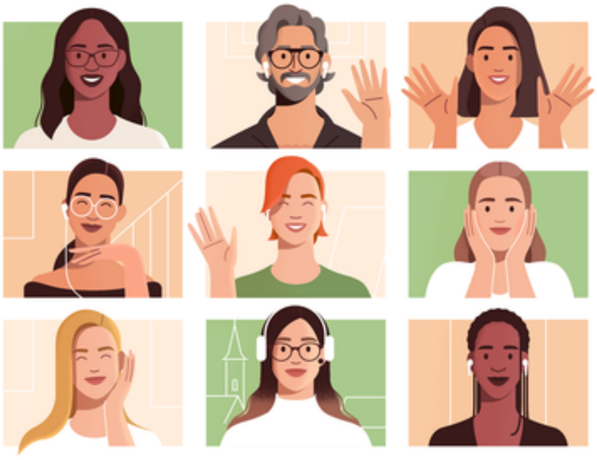

Benefits of the Virtual Community Engagement (V-CES) Model

The V-CES model provides Researchers and Community Experts an interactive, efficient, effective way to provide meaningful input into all phases of research. If conducted during the earliest stages of project development, a V-CES can be an effective tool to develop a strong partnership with community partners[7]. The virtual format of the CES enables individuals from across the United States to participate, eliminates the burden of finding transportation to a CES, saves participants' valuable time, and may reduce baby-sitting challenges. It also enables individuals to participate who otherwise would not have been able to participate if the session was conducted in person.

The V-CES provides community members the opportunity to learn not only about research and how it can benefit themselves, their families, or their community, but also to contribute to its development and execution in a way that increases Researchers'

[7] Israel et al. 2019

understanding of and sensitivity to the community and, ultimately, the value and meaning of their work. For Researchers who are not familiar with community engagement, it can open the door to a more participatory approach.

How a V-CES benefits the community:

- Community voice is heard
- Contribution to what type and how research is conducted
- Ownership of opinions and valued input
- Ability to impact the research process
- May promote the uptake of innovation

Researchers who participated in the MMHRC V-CES sessions reported that the Community Experts' feedback increased their understanding of and sensitivity to the community and informed the feasibility and appropriateness of their projects. Community Experts from the V-CESs agreed. The vast majority of Community Experts who participated in the V-CES we conducted reported that they contributed to the research projects by increasing the Researchers' understanding and sensitivity to the community.

Researchers from MMHRC V-CES sessions reported that the Community Experts' feedback increased their understanding of and sensitivity to the community and informed the feasibility and appropriateness of their projects.

2) The Virtual Community Engagement Studio Team

The V-CES is implemented by a team that includes a Community Navigator, Science Navigator, and a Manager. The team members must be knowledgeable about the research process and have experience engaging and maintaining relationships with diverse communities.

Community Navigator

The Community Navigator is a boundary spanner with familiarity with the target community, experience with academic-community partnerships, and understanding of principles of community engagement. They have experience working in diverse communities and have demonstrated a capacity to build rapport and trusting relationships with key community leaders.[8]

The Community Navigator's responsibilities include:

- Participating in the planning meeting with the CES team.
- Helping to recruit, screen, and support Community Experts who participate in the V-CES.
- Coaching the Researchers on communicating with Community Experts with personal or professional experience with maternal mental health and/or substance use.
- Identifying and building professional relationships with virtual community organizations, advocates, and social media groups who have an interest in building academic-community research partnerships.
- Developing and maintaining mechanisms to communicate with community partners, increase interaction between community partners and academic Researchers, and track the development of academic-community research partnerships.
- Supporting community education and outreach activities.

[8] Israel et al. 2019

Hiring a Community Navigator from the community puts into practice fundamental principles of community engagement such as mutual benefit, respect, and community capacity building. A respected community member is likely to have access to networks unfamiliar to someone who works in an academic setting.[9] Community Navigators may have developed skills as social workers, mental health counselors, community organizers, advocates, or community health workers, for example.

Science Navigator

The Science Navigator of the team provides guidance on identifying and recruiting Researchers to participate in the V-CES and coaches the Researchers on communicating effectively with non-Researchers and engaging Community Experts as consultants, rather than research subjects. The V-CES Team Science Navigator must have experience in patient-centered outcomes research, community-engagement, comparative effectiveness and community-based participatory research.[10]

The Science Navigator's responsibilities include:

- Participating in the planning meeting with the V-CES Team.
- Helping to identify, orient, and support Researchers who participate in the V-CES.
- Coaching the Researchers on communicating effectively with non-Researchers.
- Coaching the Researchers on engaging Community Experts as consultants.
- Developing a recommendation report from the Community Experts feedback to share with the Researcher.
- Encouraging Researchers to consult Community Experts who would like to remain involved as the research project develops.

Manager

The Manager assists with the planning and implementation of the V-CES. They work closely with the Community Navigator and Science Navigator to reach out to selected Researchers and Community Experts, scheduling the V-CES, selecting the appropriate virtual platform for the V-CES (e.g. Zoom), and preparing necessary materials. The Community Navigator or Science Navigator could take on this role if needed.

[9] Israel et al. 2019
[10] Israel et al. 2019

The Manager's responsibilities include:

- Participating in the planning meeting with the V-CES team.
- Managing logistics such as securing a technological platform and time for the V-CES that is convenient for the Community Experts across different time zones.
- Ensuring that potential participants have access to the Internet and appropriate tools and resources (e.g., laptop computer, tablet or smartphone).
- Taking notes during the V-CES.
- Monitoring participant feedback during the session and managing the participants to ensure equitable participation among those individuals who wish to actively participate during the V-CES. This will include helping the Facilitator by tracking the chat functions and checking participants' video images.
- Making sure the appropriate online surveys are completed for each V-CES, including the research project feedback and V-CES evaluation surveys, and forms needed to process payments (e.g., external consultant form and the IRS W9).

3) The Facilitator

The Facilitator's task is to create a comfortable, safe environment that allows for open and frank discussion and to guide the conversation among Researchers and Community Experts. A skilled Facilitator does not interject their opinions or biases into the conversation.[11]

The Facilitator should have experience working with mothers with mental health and/or substance use challenges and possess the ability to balance the differences in power that can naturally occur when Researchers and lay community members come together. Ideally, the Facilitator has relevant training or experience that prepares them to work with groups of individuals representing diverse social-economic, racial and disability backgrounds, with varied learning and communication styles.[12]

[11] Israel et al. 2019
[12] Israel et al. 2019

Individuals who are most successful in V-CES facilitation have been trained in the principles of community organizing or group facilitation and incorporate a participatory approach into their style of facilitation. Ideally, the V-CES Facilitator appreciates that stakeholders' perspectives are shaped by their experiences in an environment where faith, class, race, and culture play an important part in who we are and how we see the world. It is highly recommended that the Facilitator also have experience with using the selected technological platform prior to the V-CES to help prevent avoidable technological issues during the session.

The Facilitator should have experience working with mothers with experience of mental health and/or substance use challenges.

The Facilitator's responsibilities include (Israel et al., 2019)**:**

- Explaining discussion ground rules (e.g., be concise, don't interrupt, stay on track, and maintain confidentiality).
- Listening carefully to the presentation and comments, as a way to keep the discussion on track.
- Using the predefined questions as the discussion framework.
- Watching the time (1 to 1/2 -2 hours maximum) to politely move things along if someone is monopolizing the conversation, and to ensure that the two to three specific research questions/topics are addressed.
- Recognizing when more needs to be said, and in some cases, using probes and follow up questions when needed.
- Requesting examples when comments are unclear.
- Being comfortable with silence as people consider their answers.
- Guiding the discussion, only interjecting their own opinion and personal observations with intention and purpose.

In preparation for the V-CES, the Facilitator should receive and review the Facilitator V-CES Guide that includes a detailed overview of the V-CES process, a copy of the Researcher's presentation, and general background on the Community Experts. (See Appendix A.) If possible, it may be helpful for the Facilitator to attend the Researcher preparation meeting, to meet and get to know the Researcher. The Community Navigator or Science Navigator could take on this role if needed.

4) The Researcher

Researchers focused on maternal mental health and/or opioid/substance use/recovery are great candidates to apply to participate in the V-CES. In the application, Researchers describe their research and hopes for community engagement including a summary of the problem or questions they think their project will address, target population, stage of research, and questions they want to propose to Community Experts along with feedback needed. (For more details on the application and recruitment email see Appendix B.) Once selected, the Manager emails the Researcher the V-CES Researcher Guide (see Appendix C) that details the purpose and structure of the V-CES and schedules a one-hour Researcher coaching meeting about two weeks prior to the V-CES.

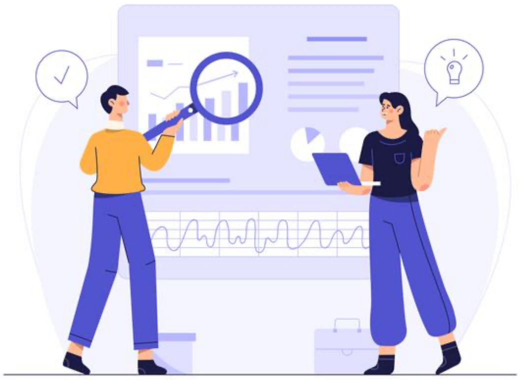

In the one-hour meeting, the V-CES team will further explain how the V-CES works and what to expect, review the Researcher's presentation and planned questions, provide guidance on communicating with the arranged Community Expert group, and discuss next steps. The V-CES team has the option to recommend changes to the presentation as needed to improve clarity and ensure the language and tone is appropriate and sensitive to the Community Experts' experiences.

Key concepts to convey when coaching Researchers (Israel et al., 2019)**:**

- The presentation should serve to elicit feedback from a group of knowledgeable consultants on how best to move forward with a research idea or project.
- The Community Experts, while not trained in research, are the experts in the room in terms of the experiences and needs of the target population.
- After making the presentation, the Researcher's role is primarily to listen, asking and answering questions for clarification.
- Researchers often use a language that non-Researchers may find difficult to understand. Jargon, technical terms, and acronyms should be explained clearly or avoided altogether. At best they are kept to a bare minimum, if used at all.

Following the initial coaching meeting the Researcher should create a very brief introduction video for the V-CES team to send to the Community Experts prior to the V-CES. The video should serve as an engaging and informal way for the Researcher to introduce themselves and why they are interested in the topic, describe the general discussion topic, and allow Community Experts to become familiar with the Researcher. The overall tone of the video should present the Researcher as an approachable coworker who is on an even power level to the Community Experts. We recommend having the Researcher use a smartphone to record their introduction video and avoid using a script or virtual background.

The Researcher should also send the V-CES their final presentation to the team. The team may need to suggest another round of edits over email to ensure the presentation is appropriate for the V-CES audience. It will also be important for the V-CES team and Facilitator to have final copies of the presentation in advance of the V-CES in case the Researcher encounters any technical difficulties during their online presentation.

The Researcher's Presentation

The presentation must be brief – no more than 10 or 15 minutes. It is important that the Researcher devote adequate time to prepare, as it can be difficult to explain research-related concepts in brief, plain language. It is not appropriate to use a presentation that was previously prepared for an academic or conference audience, unless it was adapted/modified for a lay audience. Keep text to a minimum and avoid complex tables, numbers, formulas, and diagrams.[13]

[13] Israel et al. 2019

The Researcher should prepare two to three specific questions and/or topics for the group to discuss.[14] The Facilitator will move the group discussion along to ensure that these items are all discussed.

In preparing the presentation, the Researcher must remember Community Experts need to know (Israel et al., 2019):

- What the Researcher is trying to find out, and why the Researcher thinks it is important.
- The Researcher's motivation or personal connection to the topic.
- How the planned research might impact people who would serve as research participants.
- What phase the research project is in and what kind of advice the Researcher needs.
- How the Community Experts' feedback will contribute to the research project.

5) The Community Experts

Community members, be they mothers, caregivers, peer specialists, advocates or providers are the key to the success of the V-CES. Ideally, they represent diverse backgrounds and are connected to the community in various ways.[15] Community members bring different experiences to the table and can provide insights from multiple perspectives. For example, a care manager or peer specialist who works with mothers in mental health/substance use treatment may have a very different, but equally valuable, perspective from a mother currently in recovery with a similar condition.

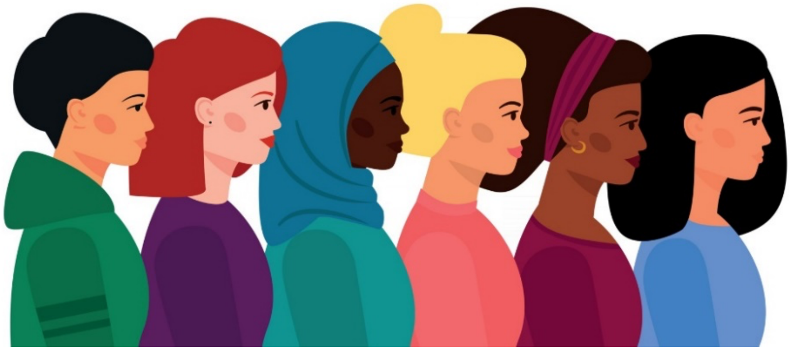

[14] Israel et al. 2019
[15] Israel et al. 2019

Generally, Community Experts should be a member of the population or community of interest and have (Israel et al., 2019):

- Good verbal communication skills.
- Good listening skills.
- Desire to learn about research.
- Willingness to share her experiences.
- Are comfortable with and have the capacity to use an online platform (e.g., Internet access, a laptop, tablet or smartphone, etc.).

The process of identifying people to serve as Community Experts will initially focus on having a diverse group of stakeholders, including those with personal and/or professional experience with maternal mental health and opioid/substance use/recovery, with an end goal of creating a core group or pool of experts familiar with and committed to the V-CES process. Researchers studying maternal mental health and/or opioid/substance use/recovery can benefit from engaging several different perspectives including: mothers who are in recovery as well as their family members, direct service providers, program planners and community organizers, and recovery coaches. It is important however, to consider how the makeup of the Community Expert panel may affect women participants who are in treatment and recovery and may have experienced trauma. For example, the Community Experts in the MMHRC V-CESs reported that they felt more comfortable sharing their experiences and opinions with the Researcher among a group of other mothers who self-reported similar experiences, rather than if we had included service providers. To get a diverse set of perspectives, you may want to hold multiple V-CESs for different stakeholders.

Researchers studying maternal mental health and/or opioid use/recovery can benefit from engaging several different perspectives.

<u>A note on working with mothers with mental health and/or opioid/substance use experience</u>: Certain research areas and topics discussed in a V-CES may be sensitive or triggering for mothers. By participating in a V-CES, mothers are choosing to engage with that content matter. It is therefore important that the Researcher and V-CES team treat the mothers (and all V-CES stakeholders) with the utmost respect and sensitivity. Additionally, the V-CES is not research, per se, and the V-CES team should not ask or

collect information beyond what is needed to ensure an appropriate, diverse group of participants.

Recruitment Process

There are numerous strategies that can be used in the recruitment process, but it is important to look beyond the "usual suspects"- those individuals who are commonly called upon to serve on Boards, Task Forces, and positions where they are asked to represent an entire community.[16] Ideally a panel of three to ten Community Experts should be present for a virtual CES. Sometimes life gets in the way, and people have to drop out at the last minute. For this reason, it is ideal to recruit a few extra people to ensure adequate participation.[17]

The V-CES format enables individuals from across the United States to participate, eliminates the burden of finding transportation to a CES, may help with the challenge of babysitting, and saves participants' valuable time. It also enables individuals to participate who otherwise would not have been able to participate in an in-person situation. The virtual format, however, may also exclude important stakeholders who do not have access to the internet, smartphone or computer, and/or do not have sufficient technical skills to navigate a virtual meeting platform.

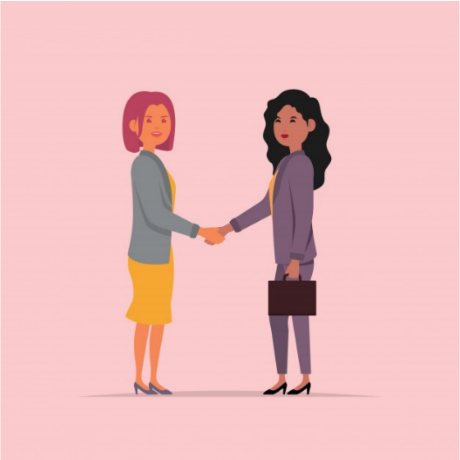

Partnering with established organizations and agencies:

In previous MMHRC V-CESs, we recruited Community Experts by reengaging partnerships with a long-standing community agency to help recruit a group of recovery coaches within their organization and with a state agency to recruit perinatal peer mentors within several statewide grant programs. This approach enabled us to reach a diverse group of Community Experts across the state who are not "usual suspects" and who have both personal and professional experience with maternal mental health issues and opioid/substance use/recovery. In addition, through these approaches, MMHRC built professional relationships with community organizations and community advocates with an interest in building academic-community research partnerships. (See Appendix D for materials for recruiting Community Experts.)

Recruiting through social media platforms:

In more recent V-CESs, MMHRC recruited Community Experts by further engaging our established Facebook groups and email listserv (see Appendix D for direct recruitment

[16] Israel et al. 2019

[17] Israel et al. 2019

email and social media post examples and tips for using social media platforms). Looking at ways to utilize social media platforms is another avenue for recruiting potential participants. Social media enables individuals from different parts of the country to participate and helps identify a robust cohort of individuals.

The V-CES team should conduct additional screening steps when using social media platforms for direct recruitment to ensure Community Experts are appropriate for the planned V-CES. The first step is including an online survey to confirm interested parties' contact information and availability as well as basic personal and/or professional mental health and/or substance use experiences to help build appropriate Community Expert groups (see Appendix F for the online survey template).

Once the V-CES team and the Researcher define the desired make up of Community Experts, the Manager or the Community Navigator contacts potential Community Experts to set up an additional screening conversation through the appropriate online platform. The purpose of this additional screening step is to confirm potential Community Experts' interest, availability, and comfort level participating in an online group discussion on how to improve research on maternal mental health and/or substance use. During this conversation it is important to explain to potential participants the purpose and procedures of the V-CES, describe the target Community Expert pool, and set expectations for next steps and participation. See Appendix D for an example topic guide for these screenings.

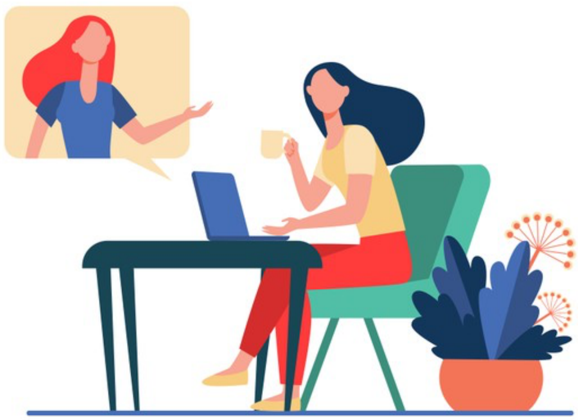

Note that part of the screening process is also ensuring that Community Experts have a relatively quiet and private place where they feel comfortable talking about sensitive topics. Community Experts should be assured that, with the intimacy of joining a virtual meeting, there will be no judgement of where the quiet place may be or looks like. For example, it is completely acceptable to join via their car, a messy kitchen table, or a family room with pets.

Compensation

The experience and knowledge that Community Experts bring to the research process are tremendous assets; they should be appropriately compensated for their time.[18]

[18] Israel et al. 2019

MMHRC provided Community Experts options between different electronic gift cards and a stipend lump sum to an agency that allowed Community Experts who were staff members to participate during regular work hours.

Community Expert Prep

Preparing Community Experts for the V-CES and buttressing their sense of agency is an important step in the V-CES process. Community Experts should come into the V-CES confident that they know what will occur during the studio, and that their questions and feedback will be heard and respected by all the participants. Even though they might not have a research background, they will be able to contribute to the research process.

Prior to the V-CES, the V-CES team should email the Community Experts: 1) the Community Expert CES Guide (Appendix E) that provides a general description of V-CES and the role of the Community Experts, and a glossary of research terms; 2) the Researcher's introduction video; and 3) the online background survey (if they did not complete it during the recruitment process). The experts can review these materials at their convenience. It is important that these materials adequately prepare the Community Experts without being overly cumbersome or time consuming, which can be a barrier to participation. Community Experts are encouraged to contact the V-CES team with any comments and/or questions.

We prepared a series of videos of Researchers addressing questions provided by mothers regarding research – Research 101 for Mothers. These are available on the MMHRC website: https://research4moms.com/research-101/. Encouraging participants to view these videos in advance of the V-CES will help them prepare.

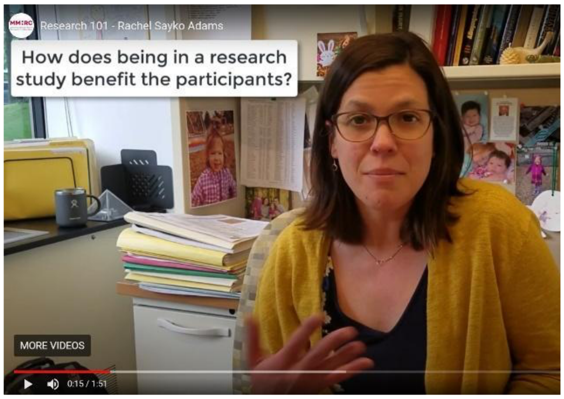

Clip from MMHRC's Research 101 videos

6) Planning and Logistics

In scheduling V-CES, it is important to choose a time that is as convenient and accessible for the Community Experts as possible. The Community Expert recruitment process will likely dictate your scheduling approach. For example, when recruiting within one partner organization it is best to have the organization set the date and time that is most convenient for the group. When recruiting through social media platforms, it is most efficient to set the date, time, and meeting platform prior to recruitment activities. The location and time zones of potential participants will likely contribute to their ability to participate. It is important to take this into consideration. The V-CES team should be cognizant that Community Experts may not have flexibility during the weekdays due to work and other conflicts, and lack of childcare may be a barrier to participation. V-CES are often held in the evenings and/or on the weekends.

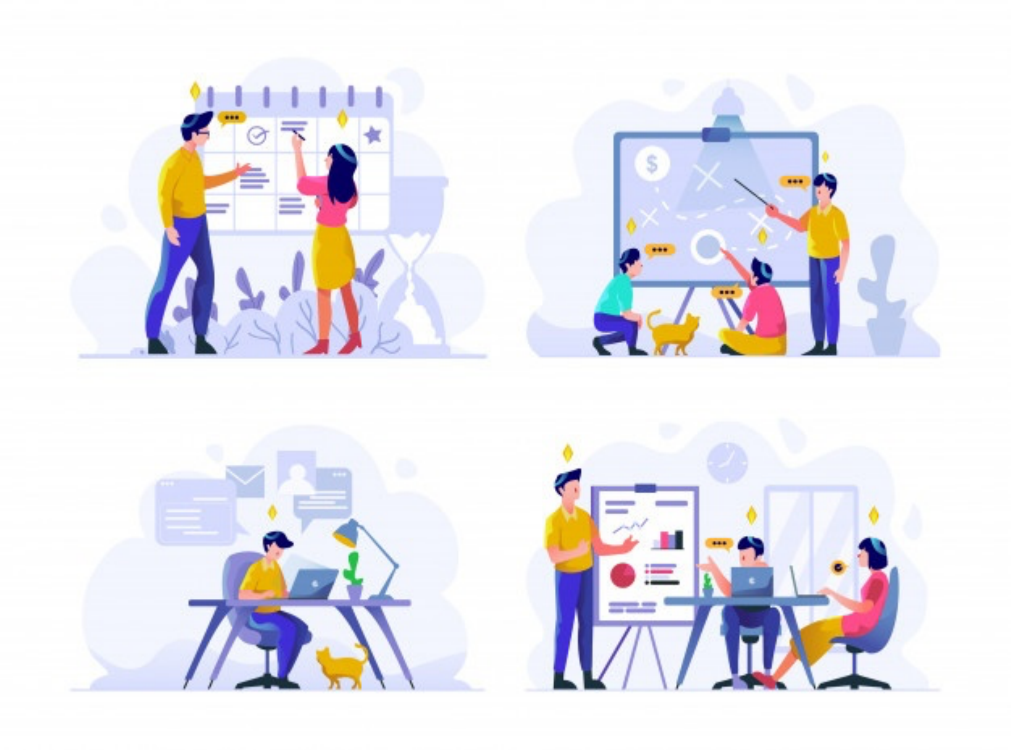

Step-by-step: Planning

Below are the outlined steps and a flow chart for preparing and hosting the overall V-CES.[19]

1) V-CES team planning meeting: Discuss goals, potential pools of Community Experts and Researchers, recruitment strategies, and outline team responsibilities.
2) Recruit Researchers to participate.
3) Recruit community members for the expert panel.
    a. Through organizations/agencies: Send contact information to distribute and recruit Community Experts.
    b. Through social media: Post to social platforms. Conduct screening virtual meetings with the potential pool of experts.
4) Email confirmed Community Experts individually to begin/continue to establish a relationship, thank them for their anticipated participation, provide them with the Community Expert Guide, send the online background survey (if applicable), and confirm next steps.
5) Email Researcher to confirm and thank them in advance for their interest in participating, send V-CES Researcher Guide, and schedule the coaching meeting with the V-CES team.
6) Secure a Facilitator for the V-CES and provide the Facilitator V-CES Guide.
7) Order and/or arrange Community Experts' and other appropriate stipends.
8) Conduct the coaching meeting with the Researcher/V-CES team to review the presentation using the technological platform that will be utilized. Make recommendations for improvement if needed. Request the final version and the Researcher introductory video prior to the V-CES.
9) Email Community Experts the Researcher introduction video.
10) Confirm time with Community Experts, the Researcher, and Facilitator. Request any outstanding items.
11) Get together appropriate online forms and materials that the V-CES team will email participants following the studio (Community Expert Research Comment Form, Community Expert V-CES Evaluation, Facilitator V-CES Evaluation, Researcher V-CES Evaluation). Adapt materials, as necessary, to overcome accessibility issues (e.g., large font for those with vision impairments).

---

[19] Israel et al. 2019

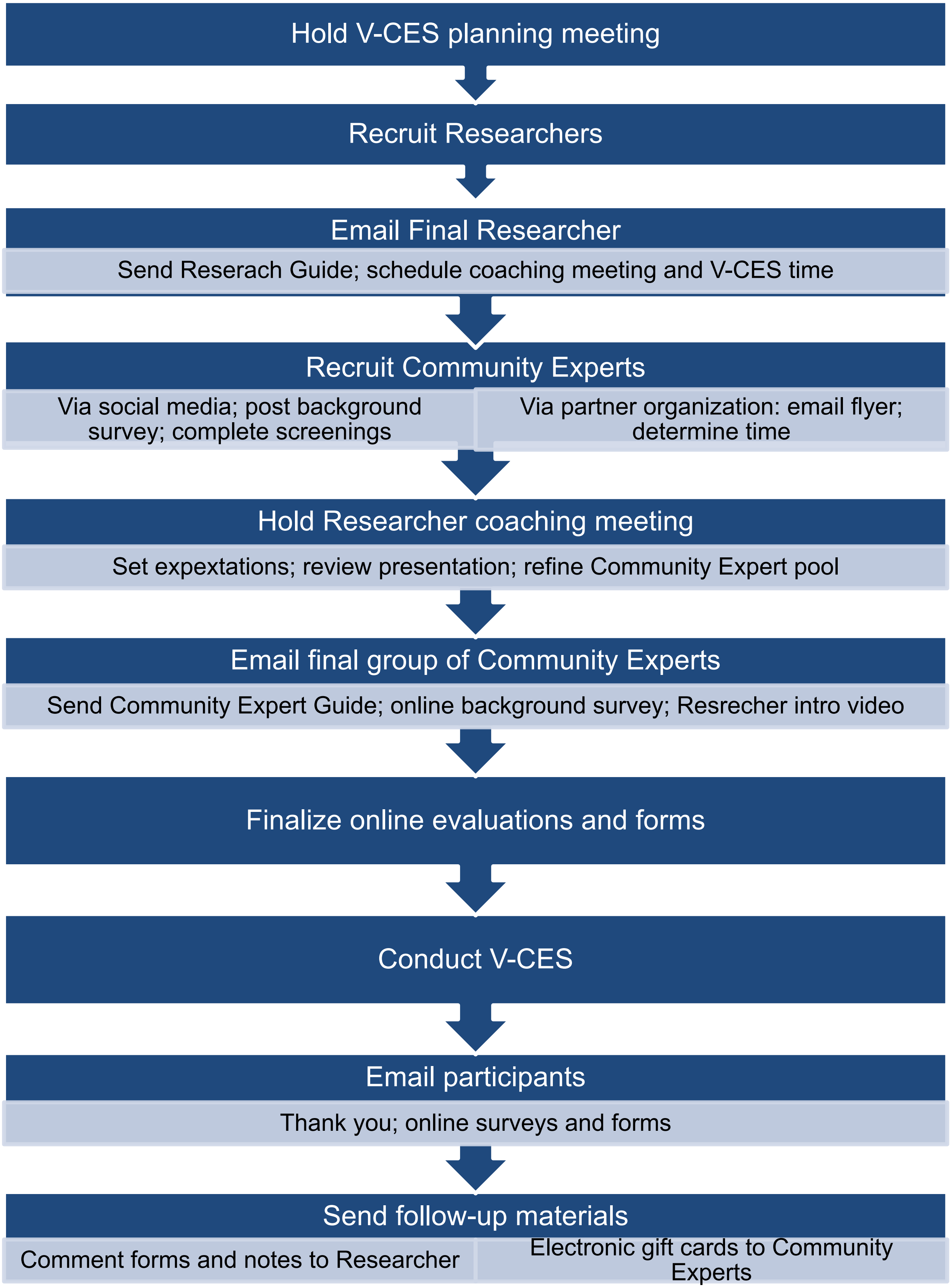
Hold V-CES planning meeting
Recruit Researchers
Email Final Researcher
Send Reserach Guide; schedule coaching meeting and V-CES time
Recruit Community Experts
Via social media; post background survey; complete screenings
Via partner organization: email flyer; determine time
Hold Researcher coaching meeting
Set expextations; review presentation; refine Community Expert pool
Email final group of Community Experts
Send Community Expert Guide; online background survey; Resrecher intro video
Finalize online evaluations and forms
Conduct V-CES
Email participants
Thank you; online surveys and forms
Send follow-up materials
Comment forms and notes to Researcher
Electronic gift cards to Community Experts

Step-by-step: Conducting the V-CES[20]

1) Facilitator and Community Navigator greet all participants warmly when they join the virtual platform.
2) When the group is all present, the Community Navigator welcomes everyone and asks them to introduce themselves briefly. Encourage participants to use Gallery View.
3) Community Navigator, Science Navigator and Facilitator introduce themselves and describe their personal/professional background to build trust with the group.
4) Community Navigator sets the context by giving a brief overview of the purpose and format of the meeting.
    a) Ground rules: be respectful of others' opinions and experiences, keep confidential any personal information that might be discussed in the meeting, stay on topic and give everyone a chance to participate.
    b) Encourage participants to keep their video on but they should feel free to turn it off if they ever feel uncomfortable or need a moment of privacy.
    c) Ask permission to record the meeting and take written notes for future use, and ensure confidentiality. Explain that it is not a problem if even one person is uncomfortable with recording the meeting or taking notes.
    d) While the Researcher is presenting, we are asking everyone to mute themselves. Participants are encouraged to make comments/questions in the chat function that will kick off the discussion following the presentation.
    e) During the discussion participants can unmute themselves to participate and can continue to use the chat and hand raise function if it is helpful.
    f) When the group discussion is over, the Manager will email the participants an online research comment sheet and V-CES evaluation.
    g) [If applicable:] The Manager will email participants an electronic gift card.
5) Researcher introduces themself and gives a brief presentation (no more than 10 or 15 minutes) describing their research project and the specific questions for the experts' consideration.
6) Community Navigator opens the conversation with questions from the chat (if applicable) and planned questions. Community Experts have the opportunity to ask researcher questions for clarification.

---

[20] Israel et al. 2019

7) Facilitator keeps the conversation on track, making sure everyone's voice is heard and that the two to three research topics or questions are addressed.
8) The Manager takes notes throughout the discussion and helps the Facilitator monitor the chat and similar functions.
9) At the end of the discussion, the Community Navigator thanks everyone for their participation and reminds participants of the next steps. The Manager will email the Researcher, Community Experts, and the Facilitator a brief online V-CES evaluation survey. See the Community Expert, Researcher, and Facilitator surveys in Appendix G. Community Experts will also receive an online Research Comment Form (see Appendix G). The Manager will email the participants online gift cards once the appropriate surveys are completed as applicable.

Costs

V-CES costs include V-CES Team time, compensation for the Community Experts, and Facilitator fees, if necessary. Including V-CES costs in grant proposals is a good idea to demonstrate active community engagement to funders.

7) Follow-up

The V-CES follow-up materials provide the Researcher important feedback from the Community Experts, allow the V-CES team to learn ways to continue to improve future V-CESs, and facilitate further collaboration between the Researcher and the Community Experts.

For the Researcher

A summary report including the V-CES team member's notes and verbatim submitted comments (de-identified, if necessary or advisable) from the Community Experts is shared with the Researcher within one week of the V-CES. The Community Expert comments should be grouped together and kept anonymous. We recommend highlighting specific recommendations as related to the topics that were discussed during the meeting.[21]

The Researcher will also receive a one-page Continuing Community Engagement Guide that suggests ways for the Researcher to maintain appropriate communication with the Community Experts who reported interest in serving as a consultant as the research project develops (See Appendix H).

For the Community Experts

[21] Israel et al. 2019

Notify them of any changes, adjustments and improvements made as a result of their input. Items shared as follow-up may include updated outreach materials, policy and procedural changes or significant accomplishments of the study due to advice received during the V-CES. If possible, give the Community Experts periodic updates on the project as well as any findings published or disseminated by the Researcher.[22] Suggest ways for Researchers to do this as well, including providing Community Experts' contact information for those who gave permission in the online evaluations.

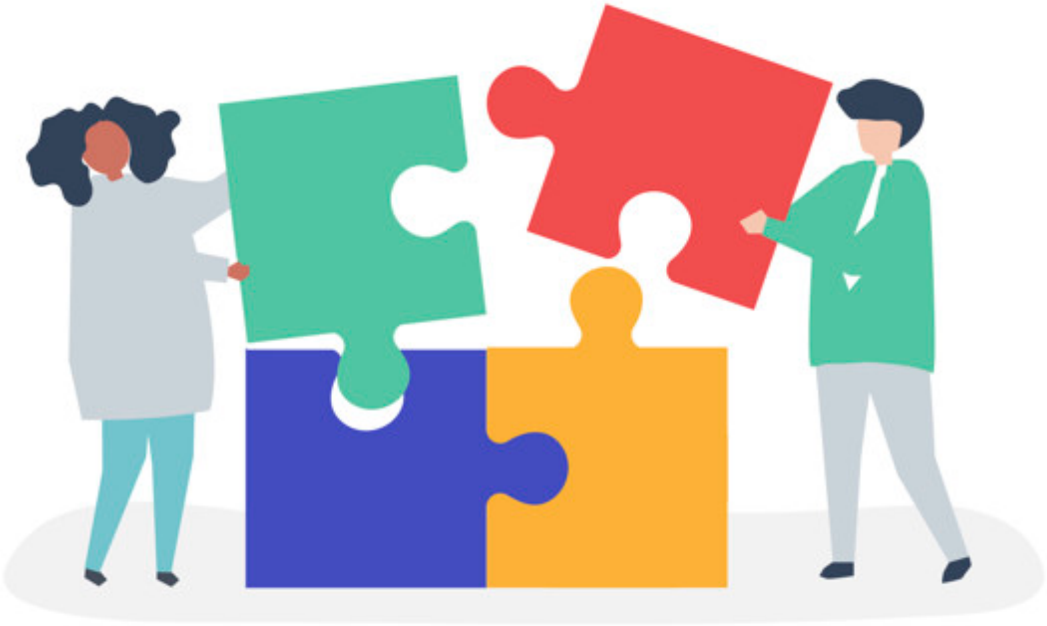

[22] Israel et al. 2019

Please use the following citation for this toolkit:

Maternal Mental Health Research Collaborative. (2021). *Virtual Community Engagement Studio Toolkit: For Researchers and Community Experts Interested in Maternal Mental Health and/or Opioid Use/Recovery*. Brandeis University Institute for Behavioral Health, Heller School for Social Policy and Management. Retrieved from https://heller.brandeis.edu/ibh/affiliates/mmhrc/index.html and http://research4moms.com.

Note: Images obtained from Google images, Pixabay Shutterstock, and Vecteezy. No copyright infringement intended.

8) References

Abu-Rus, A., Bussell, N., Olsen, D. C., Davis-Ku, M. A., Alissa L., & Arzoumanian, M. A. (2019). Informed consent content in research with survivors of psychological trauma. Ethics & Behavior, 29(8), 595–606. https://doi.org/10.1080/10508422.2018.1551802

Israel T., Farrow, H., Joosten, Y., & Vaughn, Y. (2019). *Community Engagement Studio Toolkit 2.0.* Meharry-Vanderbilt Community Engaged Research Core, Vanderbilt Institute for Clinical and Translational Research. Retrieved from https://www.vumc.org/meharry-vanderbilt/sites/vumc.org.meharry-vanderbilt/files/public_files/CESToolkit%202.0.pdf

Joosten, Y. A., Israel, T. L., Williams, N. A., Boone, L. R., Schlundt, D. G., Mouton, C. P., Dittus, R. S., Bernard, G. R., & Wilkins, C. H. (2015). Community Engagement Studios: A Structured Approach to Obtaining Meaningful Input From Stakeholders to Inform Research. *Academic Medicine*, *90*(12), 1646–1650. https://doi.org/10.1097/ACM.0000000000000794

Michener, L., Cook, J., Ahmed, S. M., Yonas, M. A., Coyne-Beasley, T., & Aguilar-Gaxiola, S. (2012). Aligning the Goals of Community-Engaged Research: Why and How Academic Health Centers Can Successfully Engage with Communities to Improve Health. Academic Medicine, 87(3), 285–291. https://doi.org/10.1097/ACM.0b013e3182441680

Spalluto, L. B., Thomas, D., Beard, K. R., Campbell, T., Audet, C. M., McBride Murry, V., Shrubsole, M. J., Barajas, C. P., Joosten, Y. A., Dittus, R. S., & Wilkins, C. H. (2019). A Community-Academic Partnership to Reduce Health Care Disparities in Diagnostic Imaging. Journal of the American College of Radiology, 16(4), 649–656. https://doi.org/10.1016/j.jacr.2018.12.033

Appendix A: Facilitator V-CES Guide

Dear Facilitator,

On behalf of the [insert organization name], thank you for participating in the upcoming virtual Community Engagement Studio (V-CES). The V-CES provides researchers and stakeholders an interactive, efficient, and effective way to engage and provide meaningful input into all phases of research. We look forward to working with you and will do our best to ensure that the V-CES is a positive and productive engagement experience for everyone.

Facilitator V-CES Guide

Overview of a Virtual Community Engagement Studio (V-CES)

The V-CES is a structured online session for Community Experts (e.g., stakeholders, including individuals with personal experience with maternal mental health issues and/or substance use, family members, practitioners, advocates, and policy makers) and Researchers to come together virtually and discuss research in a way that is culturally sensitive and honors Community Experts' priorities, values and needs.

In a V-CES, the **Researcher** gives a brief presentation about their project and poses specific questions to a group of **Community Experts**. The Researcher uses language that is appropriate and sensitive to the Community Experts' experience, background and role. The discussion that follows is guided by a **Facilitator** (you), along with the **Community Navigator**, to elicit authentic and constructive input and feedback on setting research priorities, developing and implementing a study, or disseminating research findings.

The V-CES is an interactive, consultative model; it is <u>not</u> a standing advisory board, a tool for recruiting research participants, or a data collection or research method (such as a focus group). The Community Experts who participate in the V-CES are not research subjects; rather, they are advisors and/or consultants who receive a stipend for helping with this effort.

Partnering with Mothers and Others as Community Experts

Community Experts may be mothers or mothers-to-be with past or current experience of mental health conditions and/or substance use, family members, practitioners, advocates, or policy makers. Mothers at high risk often feel judged, and mothers with experiences of mental health conditions or substance use may feel even more vulnerable. They may have experienced negative attitudes and actions of others, who make assumptions about them, perhaps informed by damaging narratives and stereotypes. Mothers may have past experiences contributing to trauma, either themselves or as the witness of bad things happening to others.

Participants who may have histories of victimization or violence may feel uncomfortable or find themselves responding in negative ways to characteristics of the V-CES or to information shared by others. Literature regarding this topic shows that participants who have experienced trauma regard the experience of sharing their stories for research positively even if it may be triggering.[23] However, it is important to be alert to this possibility and take care to guide the discussion in a sensitive and respectful manner. With this in mind, Facilitators and Researchers should refrain from asking Community

---

[23] Abu-Rus et al., 2019

Experts detailed or direct questions about their personal mental health or substance use status, beyond confirming their experience, as appropriate. If Community Experts begin to share details that may seem too personal, intimate or unsettling, the Facilitator may re-direct the conversation by kindly asking the Community Expert to suggest ways in which her experience informs the task or question at hand.

It is also possible that some Community Experts, especially those who have experienced traumatic events, may feel uncomfortable with using a video function that "exposes" or "intrudes" into their home environment. The V-CES team will inform Community Experts that participants can opt-out of the video function at any point during the V-CES. In addition, Community Experts can be encouraged to use the chat function, to type questions or responses, if they are reluctant to give voice to their perspectives or want to feel less "exposed."

Your role as the Facilitator

The Facilitator's task is to create a comfortable, safe environment that allows for open and frank discussion, and to guide the conversation between the Researcher and Community Experts. The V-CES Facilitator appreciates that stakeholders' perspectives are shaped by their experiences in an environment where faith, class, race, culture and relationships play an important part in who we are and how we see the world.

During the V-CES, the Facilitator's responsibilities include:

- Explaining discussion ground rules (e.g., be concise, don't interrupt, stay on track, and maintain confidentiality).
- Listening carefully to the presentation and comments, to keep the discussion on track.
- Using the predefined questions as the discussion framework.
- Watching the time (1 1/2 -2 hours maximum) and politely moving things along if someone is monopolizing the conversation and to ensure that the two to three specific research questions/topics are addressed.
- Recognizing when more needs to be said and using probes and follow up questions when needed.
- Requesting examples when comments are unclear.
- Being comfortable with silence as people consider their answers.
- Guiding the discussion and only interjecting your own opinion and personal observations with intention and purpose.

The V-CES Manager will provide additional support during the V-CES by tracking the chat functions and participants' videos to ensure that those who want to speak are given the opportunity to do so.

About one week before the V-CES, we will send you a copy of the Researcher's presentation and general background on the Community Experts. Please let us know if you have any questions and we thank you for participating in our V-CES.

Below is the step-by-step procedure for the V-CES.

1) Facilitator and Community Navigator greet all participants warmly when they join the virtual platform.
2) When the group is all present, the Community Navigator welcomes everyone and asks them to introduce themselves briefly. Encourage participants to use Gallery View.
3) Community Navigator, Science Navigator and Facilitator introduce themselves and describe their personal/professional background to build trust with the group.
4) Community Navigator sets the context by giving a brief overview of the purpose and format of the meeting.
    a) Ground rules: be respectful of others' opinions and experiences, keep confidential any personal information that might be discussed in the meeting, stay on topic and give everyone a chance to participate.
    b) Encourage participants to keep their video on but they should feel free to turn it off if they ever feel uncomfortable or need a moment of privacy. Note that the studio is a place of "no judgement" for at home distractions (mess, kids, pets, etc., in the background).
    c) Ask permission to record the meeting and take written notes for future use, and ensure confidentiality.
    d) While the Researcher is presenting, we are asking everyone to mute themselves. Participants are encouraged to make comments/questions in the chat function that will kick off the discussion following the presentation.
    e) During the discussion participants can unmute themselves to participate and can continue to use the chat and hand raise function if it is helpful.
    f) When the group discussion is over, the Manager will email the participants an online research comment sheet and V-CES evaluation.
    g) [If applicable:] The Manager will email participants an electronic gift card.

5) Researcher introduces themself and gives a brief presentation (no more than 10 or 15 minutes) describing their research project and the specific questions for the experts' consideration.
6) Community Navigator opens the conversation with questions from the chat (if applicable) and planned questions. Community Experts have the opportunity to ask researcher questions for clarification.
7) Facilitator keeps the conversation on track, making sure everyone's voice is heard and that the two to three research topics or questions are addressed.
8) The Manager takes notes throughout the discussion and helps the Facilitator monitor the chat and similar functions.
9) At the end of the discussion, the Community Navigator thanks everyone for their participation and reminds participants of the next steps. The Manager will email the Researcher, Community Experts, and the Facilitator a brief online V-CES evaluation survey. Community Experts will also receive an online Research Comment Form. The Manager will email the participants online gift cards once the appropriate surveys are completed as applicable.

Appendix B: Tools for Recruiting Researchers

V-CES Researcher Recruitment Invitation Template (sent via email)

Subject: An important opportunity to obtain input on your research from Community Experts

The [insert organization name] invites Researchers studying issues related to maternal mental health and substance/opioid use/recovery to apply to participate in a **virtual Community Engagement Studio (V-CES) in [insert month].** Journals and funders increasingly require investigators to provide evidence of stakeholder involvement in framing and conducting research. V-CESs provide Researchers an interactive, efficient, effective way to engage Community Experts (e.g., stakeholders including individuals with personal or professional experience with maternal mental health issues and/or opioid/substance use, practitioners, advocates, and policy makers) who can provide meaningful input into all phases of research.

In a V-CES, Researchers give a brief presentation about their project and pose specific questions to a group of Community Experts. The discussion that follows is guided by a Facilitator to elicit authentic and constructive input and feedback on setting research priorities, developing and implementing a study, or disseminating research findings.

The V-CES is an interactive, consultative model; it is not a standing advisory board, a tool for recruiting research participants, or a data collection or research method such as a focus group. The Community Experts who participate in the V-CES are not research subjects; rather, they are advisors and/or consultants who receive a stipend for their efforts.

What does participating in the V-CES entail?

1. Complete the V-CES application to provide a brief background of your research project and interest in participating in a V-CES. The application should take only a few minutes to complete.

2. If selected, you will:
    1. Prepare a brief (up to 10 or 15 minutes) presentation with questions to pose to the Community Experts.

    b. Attend a one-hour coaching meeting with the V-CES team to review your presentation and planned questions, and receive guidance on communicating with the proposed stakeholder group.

    c. Create a very brief introduction video for the V-CES team to send to the Community Experts prior to the V-CES.

d. Participate in the 1.5-to-2-hour V-CES, which will include your presentation with group discussion led by a trained Facilitator in response to your questions. Participants will complete a brief evaluation form.

e. Following the V-CES, Researchers are asked to inform the V-CES team of any changes or adjustments made to their research as a result of input from Community Experts.

f. You will have the opportunity to maintain communication with Community Experts interested in serving as consultants as your research develops. We will provide you with suggestions for the most appropriate way to build a lasting partnership with interested Community Experts.

[Insert organization name] will recruit and prepare the Community Experts and the Facilitator for the V-CES, manage all logistics (including setting up the online platform, confirming scheduling, and stipends for the Community Experts) and provide a brief report following the V-CES.

If you are interested in participating in a V-CES, please complete the initial application (see link below) by [insert date].

If you have any questions regarding the V-CES or the initial application, please contact [insert Science Navigator's name) at the [insert affiliated institution, university, or agency].

Application link: [insert application link]

V-CES Researcher Online Application Template

Thank you for applying to participate in the [insert organization name]'s virtual Community Engagement Studios. Please complete the below application.

1. Researcher/PI name
2. Researcher's contact info (phone and email)
3. Time zone
4. Researcher's background/discipline
5. Name of project
6. Summary of the problem or questions the project will address
7. Target population
8. Stage of research
9. Questions to propose to Community Experts and feedback needed

Appendix C: Researcher V-CES Guide

Dear Researcher,

On behalf of the [insert organization name], thank you for participating in our upcoming virtual Community Engagement Studio (V-CES). The V-CES provides Researchers an interactive, efficient, and effective way to engage Community Experts who can provide meaningful input into all phases of research. We look forward to working with you and will do our best to ensure the V-CES is a positive and productive engagement experience for everyone.

Researcher V-CES Guide

Overview of a Virtual Community Engagement Studio (V-CES)

The V-CES is a structured online session for Community Experts (e.g., stakeholders, including individuals with personal experience with maternal mental health issues and/or substance use, family members, practitioners, advocates, and policy makers) and Researchers to come together virtually and discuss research in a way that is culturally sensitive and honors Community Experts' priorities, values and needs.

In a V-CES, the **Researcher** (you) gives a brief presentation about their project and poses specific questions to a group of **Community Experts**. The Researcher uses language that is appropriate and sensitive to the Community Experts' experience, background and role. The discussion that follows is guided by a **Facilitator**, along with the **Community Navigator**, to elicit authentic and constructive input and feedback on setting research priorities, developing and implementing a study, or disseminating research findings.

The V-CES is an interactive, consultative model; it is not a standing advisory board, a tool for recruiting research participants, or a data collection or research method (such as a focus group). The Community Experts who participate in the V-CES are not research subjects; rather, they are advisors and/or consultants who receive a stipend for helping with this effort.

Partnering with Mothers and Others as Community Experts

Community Experts may be mothers or mothers-to-be with past or current experience of mental health conditions and/or substance use, family members, practitioners, advocates, or policy makers. Mothers at high risk often feel judged, and mothers with experiences of mental health conditions or substance use may feel even more vulnerable. They may have experienced negative attitudes and actions of others, who make assumptions about them, perhaps informed by damaging narratives and stereotypes. Mothers may have past experiences contributing to trauma, either themselves or as the witness of bad things happening to others.

Participants who may have histories of victimization or violence may feel uncomfortable or find themselves responding in negative ways to characteristics of the V-CES or to information shared by others. Literature regarding this topic shows that participants who have experienced trauma regard the experience of sharing their stories for research positively even if it may be triggering.[24] However, it is important to be alert to this possibility and take care to guide the discussion in a sensitive and respectful manner. With this in mind, Facilitators and Researchers should refrain from asking Community Experts detailed or direct questions about their personal mental health or substance use

[24] Abu-Rus et al., 2019

status, beyond confirming their experience, as appropriate. If Community Experts begin to share details that may seem too personal, intimate or unsettling, the Facilitator may re-direct the conversation by kindly asking the Community Expert to suggest ways in which her experience informs the task or question at hand.

It is also possible that some Community Experts, especially those who have experienced traumatic events, may feel uncomfortable with using a video function that "exposes" or "intrudes" into their home environment. The V-CES team will inform Community Experts that participants can opt-out of the video function at any point during the V-CES. In addition, Community Experts can be encouraged to use the chat function, to type questions or responses, if they are reluctant to give voice to their perspectives or want to feel less "exposed."

Your role as the Researcher

The V-CES requires a small investment of your time. You should expect to spend on average about four hours of your time preparing for and participating in the V-CES.

Researchers will:

- Prepare a brief (up to 15 minutes) presentation with questions to pose to the Community Experts.
- Attend a one-hour planning meeting with the V-CES team to review your presentation and planned questions and receive guidance on communicating with the proposed stakeholder group.
- Create a very brief introduction video about you and your work for the V-CES team to send to the Community Experts prior to the V-CES.
- Participate in the 1.5-to-2-hour V-CES, which will include your presentation with group discussion led by a trained Facilitator in response to your questions. Participants will complete a brief evaluation form.
- Following the V-CES, Researchers are asked to inform the V-CES team of any changes or adjustments made to their research as a result of the input from Community Experts.
- You will have the opportunity to maintain communication with Community Experts interested in serving as consultants as your research develops. We will provide you with suggestions for the most appropriate way to build a lasting partnership with interested Community Experts.

Researcher V-CES Guide – Presentation[25]

The presentation will take place on an online meeting platform and must be brief – up to 15 minutes. It is important that the Researcher devote adequate time to prepare, as it can be difficult to explain research-related concepts in brief, plain language. It is not appropriate to use a presentation that was previously prepared for an academic or conference audience, unless it was adapted/modified for a lay audience. Keep text to a minimum and avoid complex tables, numbers, formulas, and diagrams.[26]

In preparing the presentation, the Researcher must remember that in order to provide useful feedback the Community Experts will need to know:[27]

- What the Researcher is trying to find out, and why the Researcher thinks it is important.
- The Researcher's motivation or personal connection to the topic.
- How the planned research might impact people who would serve as research participants.
- What phase the research project is in and what kind of advice the Researcher needs.
- How the Community Experts' feedback will contribute to the research project.

The Researcher should prepare two to three specific questions and/or topics for the group to discuss.[28] If you haven't done so before, take time to familiarize yourself with the online platform that will be used in the V-CES. While the Facilitator will control the online operations, knowing how to share your computer screen and how basic functions work will help minimize time lost due to technological difficulties. The Facilitator will move the group discussion along to ensure that these items are all discussed.

Some researchers prefer to use PowerPoint slides for their presentation and this format can be effective in a V-CES, particularly if images or diagrams are used. However, PowerPoint slides, particularly those with a lot of text can also distract from the speaker so we recommend its use with caution. Digital handouts, prepared and emailed in advance, may be more appropriate to the situation or context. The presentation should be fairly short (no more than 5 to 10 slides) and should include:

1) Title of study, Researcher name, Researcher department/institution, date. [Note: during this slide the Researcher should describe their motivation or personal connection to the topic.]

---

[25] Israel et al. 2019
[26] Israel et al. 2019
[27] Israel et al. 2019
[28] Israel et al. 2019

2) Brief description of the purpose of the study, why the study may be important to the community, and the potential impact on/benefit to the community.
3) Study design: who, what, when, where, how.
4) How will findings be used? (dissemination/utilization plan, translation for community use).
5) Describe the current step/phase in the project and which component you need Community Experts to help inform/provide feedback. Explain how their feedback will make a difference in your research.
6) Two to three questions to propose to Community Experts and hoped-for feedback.

Researcher V-CES Guide – V-CES Agenda

Below is the step-by-step procedure for the V-CES.

1) Facilitator and Community Navigator greet all participants warmly when they join the virtual platform.
2) When the group is all present, the Community Navigator welcomes everyone and asks them to introduce themselves briefly. Encourage participants to use Gallery View.
3) Community Navigator, Science Navigator and Facilitator introduce themselves and describe their personal/professional background to build trust with the group.
4) Community Navigator sets the context by giving a brief overview of the purpose and format of the meeting.
    a) Ground rules: be respectful of others' opinions and experiences, keep confidential any personal information that might be discussed in the meeting, stay on topic and give everyone a chance to participate.
    b) Encourage participants to keep their video on but they should feel free to turn it off if they ever feel uncomfortable or need a moment of privacy. Note that the studio is a place of “no judgement” for at home distractions (mess, kids, pets, etc., in the background).
    c) Ask permission to record the meeting and take written notes for future use, and ensure confidentiality.
    d) While the Researcher is presenting, we are asking everyone to mute themselves. Participants are encouraged to make comments/questions in the chat function that will kick off the discussion following the presentation.

e) During the discussion participants can unmute themselves to participate and can continue to use the chat and hand raise function if it is helpful.

f) When the group discussion is over, the Manager will email the participants an online research comment sheet and V-CES evaluation.

g) [If applicable:] The Manager will email participants an electronic gift card.

5) Researcher introduces themself and gives a brief presentation (no more than 10 or 15 minutes) describing their research project and the specific questions for the experts' consideration.

6) Community Navigator opens the conversation with questions from the chat (if applicable) and planned questions. Community Experts have the opportunity to ask researcher questions for clarification.

7) Facilitator keeps the conversation on track, making sure everyone's voice is heard and that the two to three research topics or questions are addressed.

8) The Manager takes notes throughout the discussion and helps the Facilitator monitor the chat and similar functions.

9) At the end of the discussion, the Community Navigator thanks everyone for their participation and reminds participants of the next steps. The Manager will email the Researcher, Community Experts, and the Facilitator a brief online V-CES evaluation survey. Community Experts will also receive an online Research Comment Form. The Manager will email the participants online gift cards once the appropriate surveys are completed as applicable.

Researcher Guide – Frequently Asked Questions (FAQs)[29]

Q: What is the purpose of the virtual Community Engagement Studio (V-CES)?
A: The V-CES is a structured online session for Community Experts and Researchers to come together and discuss research in a way that is culturally sensitive and honors Community Experts' priorities, values and needs. A V-CES may be used for different types of research, including both quantitative and qualitative studies.

Q: What is the difference between a V-CES and a focus group?
The V-CES is an interactive, consultative model; it is not a standing advisory board, a tool for recruiting research participants, or a data collection or research method such as a focus group. The Community Experts who participate in the V-CES are not research subjects; rather, they are advisors and/or consultants who receive a stipend for their efforts.

Q: Why should I participate in a V-CES?

[29] Israel et al. 2019

A: Both funders and Researchers seek cost-effective and time efficient methods that engage groups of stakeholders to inform research questions, enhance research practices and improve the dissemination and use of findings. The V-CES creates a welcoming environment that works to empower community members to provide meaningful insight into all phases of research.

Q: Can I recruit experts for my study?
A: You may not use the V-CES to recruit for your study. You may, however, share information regarding the study so that a Community Expert can contact you or your research staff for more information outside of the V-CES.

Q: Why is there a Facilitator? Can't I just facilitate it myself?
A: The V-CES utilizes a trained Facilitator who has experience working with group processes and understands the principles of community engagement. The use of an independent Facilitator allows a Researcher to listen to feedback without having to worry about leading the process.

Q: Can I pick or recommend specific experts to participate?
A: The Community Navigator and the V-CES team are charged with the recruitment of Community Experts. Ideally experts should not have a prior relationship with the Researcher or the staff to ensure that feedback is open and honest. The Researcher may have relationships with community organizations or advocacy groups that may serve as recruitment sites. Once this information is provided to the team, the team can manage Community Expert recruitment.

Q: What do I need to know about mothers with mental health conditions and/or mothers who use opioids/substances in order to participate? Are there things I am not allowed to say?
A: The Community Experts may be mothers or pregnant individuals with past or current experience with various mental health conditions and/or substance use. They may also have experienced trauma. Literature regarding this topic shows that participants who have experienced psychological trauma positively regard the experience of sharing their stories for research even if it may be triggering (Abu-Rus et al., 2019). However, participants should refrain from asking the Community Experts detailed or direct questions about their mental health or substance use status, beyond confirming their experience, as appropriate, for participation in the V-CES. It is important to be thoughtful and respectful during all encounters with the Community Experts.

Q: Do I need IRB approval to participate in a V-CES?
A: The V-CES is a process to inform the development, conduct or dissemination of research but is not considered research itself. The Community Experts who participate in the V-CES are not research subjects, rather, they are advisors and/or consultants.

Q: What stage of research do I need to be in to participate? How concrete does my research project need to be?

A: We welcome applications from researchers at all stages of their research projects. The Researcher V-CES Guide provides instructions about what types of information to include in your session to describe your research.

Q: Can the information from the V-CES be used for research purposes or publication?

A: Yes and No. It is acceptable to describe the process and the input that you received, however the Community Experts are not research participants and therefore you cannot provide details of who participated or analyze their input by any demographics without express permission. You could aggregate their responses or quote them with their permission. If you have additional questions about this, ask the V-CES team.

An example of how the V-CES could be written about is below:

We received detailed input from a representative group of community experts, i.e., mothers with mental health conditions and/or mothers who use opioids, using a structured process called the Virtual Community Engagement Studio (V-CES). The V-CES uses best practices for community engagement and an experienced team to engage diverse stakeholders and facilitate meaningful discussion. When working with community experts from across the country, experts all reported feeling isolated and "unheard" by healthcare providers during the perinatal period. The V-CES sessions were important opportunities for providing these women a sense of community and agency. They wanted to continue to work with MMHRC to impact and disseminate research on maternal mental health and substance use issues.

Appendix D: Tools for Recruiting Community Experts

Partnering with established organizations and agencies:

Below is a template of the one-page recruitment flyer that we emailed to partner organizations to help them recruit potential Community Experts within their organization.

[Insert organizations name]'s Virtual Community Engagement Studio (V-CES)

Join us online on [insert date] from [insert time]

As a Community Expert, we need you now more than ever! You are one of the most important resources available to directly guide a research project and impact persons served.

What is a Community Expert? What makes me a Community Expert?

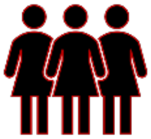

A Community Expert is someone with personal and/or professional experience with a particular community or health issue. A Community Expert has a desire to learn about research, an ability to provide input and feedback, and a willingness to be an advocate for the community.

What is a virtual Community Engagement Studio (V-CES)?

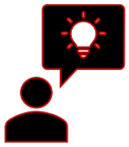

The V-CES is a structured online meeting for Community Experts and Researchers to come together and discuss research in a way that honors the priorities, values, and needs of the community.

Why should I participate?

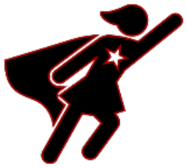

Participating in a V-CES allows you to have a direct impact on research. Your participation and unique perspective help ensure that research will be relevant and meaningful to your community.

<u>The Details:</u>

- We are inviting 10-12 Community Experts to participate in our V-CES focused on maternal mental health an opioid & substance use/recovery researcher on [insert date] from [insert time].
- You will receive a $[insert stipend amount] as a stipend for your contribution.
- You will spend about 30 minutes to read a brief guide to prepare for the V-CES and about two hours to participate in the actual V-CES session itself.
- Interested in participating or have questions? **Contact [insert Community Navigator name and email]**

Recruiting through social media platforms:

Below is an example of the content we emailed to our listserv and posted on our Facebook group to recruit directly from our community group.

SUBJECT: A- Do you want a chance to be involved in research for moms?
B - Here's your chance to be involved in research for moms

You've been waiting for it, and we're so excited to start bringing more opportunities for our partners - moms, health care providers and researchers - to get involved in our latest funded project.

The Maternal Mental Health Research Collaborative (that's us!) is currently recruiting from our community (that's you!) for participants for our upcoming virtual Community Engagement Studio on Tuesday, March 16th from 12:30pm - 2:00pm EST (9:30am - 11:00am PST).

A virtual Community Engagement Studio is:

- A chance to connect, in a small group setting, with a researcher who is looking for feedback and input into a research project about maternal mental health and/or substance use.
- An opportunity to influence how a research project is created, conducted, and completed.
- A platform to raise your voice and share your experience.

What you need to know:

- We're looking for a diverse group of women with personal and/or professional experience with maternal mental health issues and/or substance use/recovery.
- Participation is completely voluntary.
- The Community Engagement Studio will take place online, using Zoom.
- We'd like you to read a few brief documents before the Community Engagement Studio session.
- The Community Engagement Studio will be about 1 to 1.5 hours long.
- You will receive a **gift card** for your participation.

Interested?

Click here to take our quick survey.

Once you've done that, we'll review your response and be in touch within a few days.

Don't miss this opportunity to explore, share and contribute to maternal mental health/substance use research!

If you have any questions, please email mmhrc@brandeis.edu.

Below is an example outline for Community Expert initial screening.

1. Thank potential participant for their interest and time.
2. Purpose of this conversation is to describe the V-CES in more detail, answer questions, and make sure it's a good fit from the perspective of the participant.
3. V-CES purpose and potential topics and themes:
    1) The V-CES model provides a framework for connecting researchers and community members in a way that is sensitive to background, culture and community.
    2) Provides community members the opportunity to inform next steps in research.
4. V-CES procedure and expectations:
    1) Prep work: If a good fit, will email short guide and researcher introductory video prior to V-CES.
    2) Describe characteristics of target sample of Community Experts.
    3) During V-CES:
        - Researcher, 8-10 Community Experts, V-CES team come together through virtual platform.
        - Researcher describes study and asks questions in 10 min presentation.
        - Open discussion. Not group therapy, using experience to inform / improve research.
        - Participants are respectful, kept confidential.
    4) After V-CES:
        - Complete brief online surveys.
        - Emailed electronic gift card.
        - Able to continue to work with Researcher if want to.
5. Answer questions.
6. Confirm availability and comfort with the opportunity and the procedures.
    1) Time, schedule.
    2) Quiet place to be to talk about sensitive issues, non-judgmental.
    3) Able to use virtual platform.
7. Review next steps, how/when reach out

Appendix E: Community Expert V-CES Guide

Dear Community Expert,

On behalf of the [insert organization name], thank you for agreeing to serve as a Community Expert for the virtual Community Engagement Studio (V-CES). The V-CES provides Researchers an interactive, efficient, and effective way to engage people who can provide meaningful input based on their own experiences into all phases of research. The V-CES provides you the opportunity to not only learn about research and how it can benefit you, your family and community, but also to contribute to its development and implementation. Our goal is to increase the Researchers' understanding of, and sensitivity to the community and what makes the findings most relevant.

We appreciate your participation as an expert because of your knowledge of maternal mental health and/or substance use/recovery. The V-CES is an online forum for learning for both Researchers and Community Experts. We encourage you to offer honest feedback, constructive criticism, and serve as an advocate for your community.

We look forward to working with you and will do our best to ensure that the V-CES is a positive and interesting experience for everyone!

Community Expert V-CES Guide – Frequently Asked Questions[30]

Q: What is the purpose of the Community Engagement Studio (CES)?
A: The V-CES is a structured online session for Community Experts and Researchers to come together and discuss research in a way that is culturally sensitive and honors Community Experts' priorities, values, and needs. A V-CES typically takes about 1-1/2 hours to allow for a brief presentation from a Researcher and an interactive discussion, with everyone participating.

Q: What is a Community Expert?
A: A Community Expert is an individual who has experience or possesses first-hand knowledge of a community or health issue. For our purposes, we are looking for individuals with personal or professional experience with maternal mental health and/or substance use/recovery. A Community Expert has a desire to learn about research, an ability to provide constructive input or feedback, and a willingness to be an advocate for their community.

Q: Why are virtual Community Engagement Studios important?
A: V-CES help assure that research meets the needs of people who live in a community or are affected by a specific health issue. Community Experts are one of the most important resources available to directly guide a research project. Through the V-CES, the Community Expert:

- Provides the Researcher with a deeper understanding of and sensitivity to persons served and their unique circumstances.
- Helps assess the relevance, feasibility, and appropriateness of the research activities.
- Engages the community in developing research questions and considering the best ways to find answers.
- Enables community voices to be heard and impact the research process as well as the eventual uptake of research findings.

Q: Is it ok if I don't know anything about research?
A: You do not need to know anything about research in order to be a Community Expert. We just ask that you be yourself and share your thoughts and opinions based on your experience. We are providing you a glossary of some research terms and several short Research 101 videos [see more details below] as helpful resources you can review before the V-CES.

Q: How do I prepare for a CES?

---
[30] Israel et al. 2019

A:

- Please finish reviewing this guide.
- [If not already completed during the screening process:] Please complete the brief online survey that asks for your general background information (we emailed you a link to the survey). The survey should only take a few minutes. The background information you provide will be kept confidential.
- Please watch the short intro video from, [insert name], the researcher who will be asking for your feedback during the V-CES (we emailed you video). The video will help you get to know the researcher and what they plan to discuss during the studio.
- Please make sure you have [insert platform name] downloaded on your computer or phone and that you are able to login. If you have any questions or problems with [insert platform name], please email [insert manager contact information]. [She/he/they] will help you set everything up to make sure you are ready to go before the V-CES.
- Please make sure you have a relatively quiet and private place where you feel comfortable talking about sensitive topics. Do not worry, there will be no judgement of where the quiet place may be or looks like. For example, it is completely acceptable to join via your car, a messy kitchen table, or a family room with pets. It might be hard to participate if the television is loud, however.
- If you would like to learn more about research prior to the CES, the Maternal Mental Health Research Collaborative (MMHRC) created a series of Research 101 videos as an additional reference. You can find these videos on the MMHRC website: https://research4moms.com/research-101/.
- There is no need for additional preparation. You are participating in this V-CES because of your experience, background, and knowledge. Remember, you are already an expert!

Q: What can I expect?
A:

- The V-CES will last no more than 1-1/2 to 2 hours. After introductions, the Researcher will give a brief overview of their project and then pose specific questions for you to consider to help inform and/or improve the research project. The Researchers value your advice, opinions, and experience and would like to incorporate your feedback into their projects.
- Following the short presentation, the rest of the V-CES will be a group discussion around the two to three questions the Researcher poses to the group. A Facilitator will help moderate the discussion to make sure everyone has a chance to be heard. You can always type your opinions or responses into the Chat box.

- The V-CES is a group experience, even though it's not group therapy. We ask all participants to respect each other and to keep confidential any comments made during the V-CES by either the other Community Experts or the presenting Researchers.
- At the close of the V-CES, we will email you a brief online comment form for the Researcher and V-CES evaluation survey that will allow you to give additional feedback that might be helpful to the Researcher and future V-CESs.
- If you indicate that you would like to continue as an expert, the V-CES team or the Researcher may contact you in the future to get additional feedback on and/or help with other parts of the research project. You can of course decline to participate at any time.

Q: Do I have to share any personal information?
A: The V-CES is not a research study or a group therapy session. The Researcher will not ask personal questions about your life. The questions asked during the V-CES will be about your thoughts and opinions about the presented research. Should you desire to share personal information during the V-CES, please focus on how your experience might inform the research being discussed. We ask all participants to keep the shared information confidential.

Q: Will I get paid for my time?
A: Yes. We will provide Community Experts a [insert stipend amount and format, e.g., gift card] as a stipend for their contributions.

Glossary of Research Terms[31]

Data Analysis: The process of displaying, describing, summarizing, and looking for relationships among data.

Data Collection: The process of collecting information to answer research questions. Common data collection methods include gathering information from available resources (ex: hospital treatment costs or patient service uptake), making observations, conducting individual or group interviews, and administering surveys.

Data Interpretation: Drawing conclusions and meaning from the findings and results.

Dissemination: A planned process for stakeholders who could benefit from the research and those who could make real-world changes based on the research.

Patient-Centered Outcomes Research (PCOR): Helps people and their caregivers communicate and make informed health care decisions, allowing their voices to be heard in assessing the value of health care options. This research aims to answer patient-centered questions as defined by individual patients.

PI or Principal Investigator: The Researcher in charge of a research project and the person responsible for the overall management and direction of the project.

Recruitment and Retention: How Researchers reach out to the group of people they are interested in studying and get them to agree to participate and stay in a research project.

Research Design: The overall strategy the Researchers may use to answer their research questions. Some research designs describe the current landscape of an important issue (e.g., the number of women in MA who have used opioids before and after pregnancy). Other research designs try to explain and make predictions about how introducing something new to an existing issue will make a change (e.g., adding peer specialist services to existing opioid treatment programs). The research design will influence what, how, and when the Researcher will measure, collect, and analyze data.

Stakeholders: Individuals, organizations or communities that have a direct interest in the process and outcomes of a research project. Stakeholders may be patients, caregivers, practitioners, advocates, research volunteers or community leaders. The target stakeholders for the CES are individuals with experience with a particular health issue. We refer to stakeholders as “experts” and each is compensated for participation.

[insert organization name]: [insert organization background]

---

[31] These terms are adapted from the original Vanderbilt CES toolkit; Israel et al. 2019.

Appendix F: Community Expert Initial Background Online Survey

We are very excited that you are interested in joining the Maternal Mental Health Research Collaborative's (MMHRC) Virtual Community Engagement Studio. We are asking all interested candidates to please complete this initial survey so we can learn a little bit more about you. All of your answers will be kept confidential within our small MMHRC group. At the end of the survey, there is a place to ask us any questions you may have about the project.

1. Please provide us some contact information for the best way to reach you moving forward.
    a. Name:
    b. Email address:
    c. Phone number:

2. Please provide us with the following general information about yourself.
    a. Gender:
    b. Age:
        - o 18 - 25
        - o 25 - 35
        - o 35 - 45
        - o 45 - 55
        - o 55 and over

3. Do you live in the US or Canada?
    - o US
    - o Canada

4. State/providence where you live:

5. Race/Ethnicity: (check all that apply):
    - o White
    - o Black or African American
    - o American Indian or Alaska Native
    - o Asian
    - o Native Hawaiian or Pacific Islander
    - o Hispanic/Latino
    - o Other (please describe):

6. We also hope to create a Virtual Community Engagement Studio with a diverse range of personal and professional experience. Please indicate the areas you identify with and/or have personal or professional experience with (check all that apply). Your information will be kept confidential. We will not share this information with any of the other participants or anyone outside of the MMHRC staff.

- Motherhood and/or pregnancy
- Opioid use
- Cannabis use
- Alcohol use
- Nicotine use
- Cocaine use
- Other drug/substance use
- In recovery
- Perinatal mental health challenges
- General mental health challenges
- Health advocate
- Policy maker
- Healthcare provider
- Peer Specialist/Recovery Coach
- Person served/consumer
- Behavioral health clinician or counselor
- Other (please describe):

Now we would like to learn a little bit more about your access to technology and availability.

7. Do you have access to a computer, tablet, or smartphone to participate in an online meeting?
    - Yes
    - No

8. Do you have experience using Zoom or other online meeting platforms? (Don't worry, MMHRC can help you use Zoom if you need support)
    - Yes
    - No

9. Please confirm that you are available on Tuesday, March 16th from 12:30pm - 2pm EST (9:30am - 11am PST).
    - Yes, I am available.
    - No, I am not available, but I am interested in participating in a future virtual Engagement Studio.

10. Feel free to include any questions you have about the virtual Community Engagement Studio. We will answer your questions as soon as possible.

Thank you for completing this survey and your interest in participating in MMHRC's Virtual Community Engagement Studio. [If applicable:] Someone from our team will email you in the next few days to follow up.

Appendix G: Participant V-CES Forms and Surveys[32]

Community Expert Research Comment Form

Researcher:____________________________ [Prepopulate]

Thank you for participating in [insert organization] virtual Community Engagement Studio (V-CES). We'd appreciate your anonymous feedback on the Researcher's project.

Please be as detailed as possible. Your feedback is crucial to furthering the quality of the research discussed during the V-CES.

1. What did you think was good about the research idea(s)?

2. What challenges do you think a research project like this will have in the community?

3. What would you like to see the Researcher do differently or add to the project?

4. Do you have any additional thoughts about the ideas or the proposed project?

Thank you for completing this form and for participating in [insert organization]'s Virtual Community Engagement Studio.

[32] These questions are adapted from the original Vanderbilt CES toolkit; Israel et al. 2019

Community Expert V-CES Evaluation Survey

Thank you for participating in [insert organization name]'s virtual Community Engagement Studio. Please complete the following survey to help improve the effectiveness of the virtual Community Engagement Studio and the quality of the academic-community research partnerships that result. Your feedback will be kept anonymous, and your participation is appreciated.

Please tell us whether you agree or disagree with the following statements:

1) The scheduling/communications for the Studio session were handled in a timely and efficient manner.
   - Strongly agree
   - Agree
   - Disagree
   - Strongly disagree

2) The allotted time for the Studio was sufficient.
   - Too much time
   - Enough time
   - Not enough time

3) The Facilitator managed the allotted time sufficiently and addressed my questions and comments.
   - Strongly agree
   - Agree
   - Disagree
   - Strongly disagree

4) I felt adequately prepared for the V-CES.
   - Strongly agree
   - Agree
   - Disagree
   - Strongly disagree

5) The Researcher's presentation was easy to understand and gave me enough information to provide appropriate feedback.
   - Strongly agree
   - Agree
   - Disagree
   - Strongly disagree

6) Would you participate in a Community Engagement Studio again?
    - o Yes
    - o No

7) What do you feel was **your** contribution to the research project? Please check all that apply.
    - o Increased the Researcher’s understanding of the community
    - o Increased the Researcher’s sensitivity to the community
    - o Gave feedback on the feasibility of the project
    - o Gave feedback on the appropriateness of the project
    - o Provided ideas on recruiting research participants
    - o Provided ideas on research procedures, e.g., questions to ask and how to ask them
    - o Provided ideas on how to inform the community about the project
    - o Provided ideas on how to use results of project to benefit the community
    - o □ Other (please describe):

8) Please suggest at least one way the virtual Community Engagement Studio could be improved in the future:

9) Can the Researcher(s) contact you sometime in the future for your feedback on other parts of the project as the research moves forward?
    - o Yes
    - o No

    If yes, please provide the best way to contact you: ________________

Thank you for completing this survey and for participating in [insert organization]’s virtual Community Engagement Studio.

Researcher V-CES Evaluation Survey

Thank you for participating in [insert organization name]'s virtual Community Engagement Studio. Please complete the following survey to help improve the effectiveness of the virtual Community Engagement Studio and the quality of the academic-community research partnerships that result. There are no known risks to completing this survey, and your participation is voluntary and greatly appreciated.

Please tell us whether you agree or disagree with the following statements.

1) The scheduling/communications for this Studio session were handled in a timely and efficient manner.
    - Strongly agree
    - Agree
    - Disagree
    - Strongly disagree

2) The allotted time for the Studio was sufficient.
    - Too much time
    - Enough time
    - Not enough time

3) I felt adequately prepared for the V-CES.
    - Strongly agree
    - Agree
    - Disagree
    - Strongly disagree

4) The Facilitator effectively managed Community Expert feedback.
    - Strongly agree
    - Agree
    - Disagree
    - Strongly disagree

5) The Community Experts provided me with valuable feedback.
    - Strongly agree
    - Agree
    - Disagree
    - Strongly disagree

6) This Studio session will improve my research.
   - o Strongly agree
   - o Agree
   - o Disagree
   - o Strongly disagree

7) I would recommend a virtual Community Engagement Studio session to a colleague.
   - o Yes
   - o No

8) If relevant to your research, would you request a virtual Community Engagement Studio in the future.
   - o Yes
   - o No

9) Would you request input again on your current research from the individuals who participated in the Studio?
   - o Yes
   - o No

10) What do you feel were the Studio experts' contribution to the research project? Please check all that apply.
   - o Increased my understanding of the community
   - o Increased my sensitivity to the community
   - o Gave feedback on the feasibility of my project
   - o Gave feedback on the appropriateness of my project
   - o Provided ideas on recruiting research participants
   - o Provided ideas on how to inform the community about the project
   - o Provided ideas on how to use the results of my project to benefit the community
   - o Other (please describe:)

11) Has your perception about the role of patient or community stakeholders in research changed as a result of the Community Engagement Studio?
   - o Yes
   - o No

If yes, how has it changed?

12) What do you plan to change as a result of the feedback you received from the Studio? (Please check all that apply)
   - Research question
   - Research design
   - Level of community / patient engagement in research activities
   - Recruitment/retention strategies
   - Consent process
   - Data collection
   - Data interpretation
   - Dissemination
   - Change in number of questions (i.e. survey items)
   - More patient-centered questions
   - Less technical/medical jargon
   - More culturally relevant questions
   - Other (Please describe:)
   - I do not intend to change anything

13) To what degree has the Community Expert input you received from the session impacted the community or patient-centered components of your project? This includes community or patient preferences, needs, wants and values.
   - No impact
   - Minor impact
   - Moderate impact
   - Major impact

Thank you for completing this survey and for participating in [insert organization]'s virtual Community Engagement Studio.

Facilitator V-CES Evaluation Survey

Thank you for participating in [insert organization name]'s virtual Community Engagement Studio. Please complete the following survey to help improve the effectiveness of the virtual Community Engagement Studio and the quality of the academic-community research partnerships that result. Your participation is voluntary and greatly appreciated.

Please tell us whether or not you agree or disagree with the following statements.

1) The scheduling/communications for this Studio session were handled in a timely and efficient manner.
    - Strongly agree
    - Agree
    - Disagree
    - Strongly disagree

2) The allotted time for the Studio was sufficient.
    - Too much time
    - Enough time
    - Not enough time

3) I felt adequately prepared for the V-CES.
    - Strongly agree
    - Agree
    - Disagree
    - Strongly disagree

4) The relevant experts were present at the Studio session.
    - Strongly agree
    - Agree
    - Disagree
    - Strongly disagree

5) Would you recommend a virtual Community Engagement Studio session to a colleague?
    - Yes
    - No

6) Would you facilitate a virtual Community Engagement Studio in the future?

- Yes
- No

Thank you for completing this survey and for participating in [insert organization]’s virtual Community Engagement Studio.

Appendix H: Continuing Community Engagement Tips for Researchers

Consultants, Not Research Subjects
Researchers have the opportunity to move beyond the one-sided approach of learning from Community Experts' experience as a knowledge development effort that informs your general understanding of an issue. Instead, we encourage you to continue to engage Community Experts as knowledgeable consultants who can provide you insightful, tangible advice on important components of your research project.

For example, Community Experts can contribute to research projects by:

- Prioritizing research topics
- Increasing the relevance to communities
- Strengthening recruitment and retention efforts
- Helping frame, review, or develop protocols and procedures
- Participating in data collection and analysis
- Assisting with data interpretation
- Increasing dissemination and uptake of research results

As consultants, Community Experts' voices should carry the same weight as other academic consultants and they should be appropriately compensated for their contributions and time.

Sensitivity and Respect
Certain research areas and topics may be sensitive or triggering for mothers with personal and/or professional experience with maternal mental health issues and/or substance use/recovery. Therefore, it is important to remember that:

- In initial conversations with the Community Experts, you should state that you may discuss sensitive topics and that not only are the experts not required to share any personal experiences, but they can also choose to not participate at any time.
- Researchers should treat the Community Experts with the utmost respect and sensitivity.
- It is inappropriate and unethical to collect unnecessary or excess information pertaining to an individual's background, mental health, or substance use.

Convenient and Accessible

- Schedule phone and/or virtual meetings during times that are most convenient to the Community Experts. This may be after work hours, on weekends or when childcare is available.

- If asking Community Experts to review materials or complete surveys, consider that they may not have access to printers or computers outside of work. Online surveys and communications should be smartphone compatible. Again, surveys should be regarding concrete advice on your project, not as a general data collection effort.
