# Supplementary material for: Virtual Community Engagement Studio (V-CES): Engaging Mothers With Mental Health and Substance Use Conditions in Research
Source: Front Psychiatry. 2022 Jun 15;13:805781. doi: 10.3389/fpsyt.2022.805781 (PMC9240264; doi:10.3389/fpsyt.2022.805781)
Supplement: Supplementary file 2 [file Data_Sheet_2.PDF]

Sharing Research Findings with Mothers

Mothers are busy. Here are some tips to reach them effectively.

Start with the most important takeaway message.
Highlight key points.

Connect to existing knowledge.
Start with familiar ideas to build on what mothers already know.

Use examples to describe complex ideas.
Provide anecdotes or references to everyday life.

Describe the potential impact.
Suggest ways findings can inform mothers' lives and decisions.

Avoid jargon.
Replace scientific terms with everyday words.

Check the reading level.
Use 6th to 8th grade reading levels for maximum clarity.

Keep it brief.
Summarize your message in easy-to-read bullet points.

Be respectful.
Speak to mothers as you would a friend or neighbor.

MM:RC
Maternal Mental Health
Research Collaborative

Brandeis | THE HELLER SCHOOL FOR SOCIAL POLICY AND MANAGEMENT
Institute for Behavioral Health

research4moms
research4moms
research4mom
research4moms.com
heller.brandeis.edu/ibh/affiliates/mmhrc
linkedin.com/company/25065411

Funded through Patient-Centered Outcomes Research Institute® (PCORI®) Eugene Washington PCORI Engagement Awards (#8285-BU and #EAIN-00147). The views presented here are solely the responsibility of the author(s) and do not necessarily represent the views of PCORI®, its Board of Governors or Methodology Committee. #1 © 2021 MMHRC

Mothers and Trauma-Informed Research

Many mothers with mental health and substance use conditions have had traumatic experiences. Here are some tips for engaging them in research, keeping this in mind.

Provide Choices. Schedule sessions at mothers' convenience, in modes they prefer (e.g., in-person, telephone, video conference).

Be empathic. Ensure that if mothers feel uncomfortable about questions, they can choose not to answer, take a break, or stop participating at any time.

Be respectful. Take a non-judgmental stance. Listen, but don't make assumptions. A mother's way of evaluating or making meaning of an experience may not be the same as yours.

Offer privacy. Ask about mothers' preferred mode of communication. Is it OK to leave a voice or email message?

Have resources identified. Specify a safety plan in your protocol, and suggest follow-up resources, if warranted.

MM:RC
Maternal Mental Health Research Collaborative

research4moms.com

Brandeis | THE HELLER SCHOOL FOR SOCIAL POLICY AND MANAGEMENT Institute for Behavioral Health

heller.brandeis.edu/ibh/affiliates/mmhrc

research4moms

research4moms

research4mom

linkedin.com/company/25065411

Funded through Patient-Centered Outcomes Research Institute® (PCORI®) Eugene Washington PCORI Engagement Awards (#8285-BU and #EAIN-00147). The views presented here are solely the responsibility of the author(s) and do not necessarily represent the views of PCORI®, its Board of Governors or Methodology Committee. #2 © 2021 MMHRC

Participating in Research: What Do Mothers Want to Know?

Mothers want to participate in research. Their diverse backgrounds and varied experiences affect their decision to participate. Address common questions and concerns in an approachable, friendly way.

What's in it for you? Communicate clearly what your study's about and what you hope to achieve.

What's in it for them? Say how mothers' participation will make a difference, and whether they will receive a gift card or stipend. Give them choices, if possible.

What do you expect? Describe the time and tasks involved.

Who will see their information? Tell mothers who will access the data and how it will be kept confidential.

What if mothers are hesitant or uncomfortable? Explain that participation is voluntary and that they are free to leave the study at any time.

Why do you care? Share your motivation – professional or personal - if appropriate.

MMHRC
Maternal Mental Health Research Collaborative

Brandeis | THE HELLER SCHOOL FOR SOCIAL POLICY AND MANAGEMENT
Institute for Behavioral Health

research4moms.com

heller.brandeis.edu/ibh/affiliates/mmhrc

research4moms

research4moms

research4mom

linkedin.com/company/25065411

Funded through Patient-Centered Outcomes Research Institute® (PCORI®) Eugene Washington PCORI Engagement Awards (#8285-BU and #EAIN-00147). The views presented here are solely the responsibility of the author(s) and do not necessarily represent the views of PCORI®, its Board of Governors or Methodology Committee. #3 © 2021 MMHRC

Engaging Mothers Through Social Media

Social media can be effective in engaging mothers who are interested in research. These tips will help your efforts on Facebook, Instagram, and YouTube.

- Experiment with each platform to learn how it works before creating content.
- Find and join an active community of social media users with an interest in your topic.
- Tailor content for each platform. The message may be similar, but the way it's worded or how it's shared should differ.
- Test versions of posts on each platform to see how they perform. All platforms provide you with data to help with this.
- Use plain, everyday language and avoid jargon.
- Include relevant, engaging visual images with your text. You can explore royalty-free images at www.unsplash.com and www.pexels.com
- Research which hashtags are most relevant to mothers you hope to engage. Hashtags are essential to any effective social media post. Common hashtags used by mothers with maternal mental health issues include: #postpartumdepression #postpartumanxiety #ppd #ppa #maternalmentalhealth #postpartumrecovery #realmotherhood.

MMHRC
Maternal Mental Health Research Collaborative
research4moms.com

Brandeis
THE HELLER SCHOOL FOR SOCIAL POLICY AND MANAGEMENT
Institute for Behavioral Health
heller.brandeis.edu/ibh/affiliates/mmhrc

research4moms
research4moms
research4mom
linkedin.com/company/25065411

Funded through Patient-Centered Outcomes Research Institute® (PCORI®) Eugene Washington PCORI Engagement Awards (#8285-BU and #EAIN-00147). The views presented here are solely the responsibility of the author(s) and do not necessarily represent the views of PCORI®, its Board of Governors or Methodology Committee. #4 © 2021 MMHRC

Recruiting Mothers as Partners and Participants in Research

Mothers want to contribute to research. Consider these points in planning your recruitment approach.

Highlight incentives. Lead with how mothers' participation will have an impact and whether they will receive a gift card or stipend for their efforts.

Keep it clean and simple. Use plain language in bulleted text to make materials easy to read and understand.

Make it accessible. Create materials in multiple formats (e.g., printable text, videos, photos) to recruit through social media, email, and paper flyers.

Inspire curiosity. Include relevant and engaging infographics, memes, images, and videos.

Follow up. Respond quickly to mothers who express interest. Connect directly in a phone call or email to let them know next steps.

MMHRC
Maternal Mental Health Research Collaborative
research4moms.com

Brandeis
THE HELLER SCHOOL FOR SOCIAL POLICY AND MANAGEMENT
Institute for Behavioral Health
heller.brandeis.edu/ibh/affiliates/mmhrc

research4moms
research4moms
research4mom
linkedin.com/company/25065411

Funded through Patient-Centered Outcomes Research Institute® (PCORI®) Eugene Washington PCORI Engagement Awards (#8285-BU and #EAIN-00147). The views presented here are solely the responsibility of the author(s) and do not necessarily represent the views of PCORI®, its Board of Governors or Methodology Committee. #5 © 2021 MMHRC

Engaging Mothers Through Facebook

Facebook is the world's largest social media platform with over 2 billion users. Follow these tips to engage mothers in research.

Create a Facebook page for your research project. Describe yourself, your work, and what you hope to achieve using simple, conversational language.

Explain why you need mothers to participate. Focus on what's in it for them or how being involved might help others.

Make the information you share as clear, detailed, and specific as possible. This helps Facebook push your content to the appropriate audience.

Post photos or create short videos using a smartphone to share. Facebook users prefer videos.

Post a "call to action" letting mothers what you'd like them to do next. For example, mothers could:

- Comment on your post and share it with friends.
- Sign up for a newsletter or visit your blog.
- Contact you to request more information about your study.

Respond to comments and questions as soon as possible to encourage engagement and sustain interest.

MM:RC Maternal Mental Health Research Collaborative

research4moms.com

Brandeis — The Heller School for Social Policy and Management, Institute for Behavioral Health

heller.brandeis.edu/ibh/affiliates/mmhrc

research4moms

research4moms

research4mom

linkedin.com/company/25065411

Funded through Patient-Centered Outcomes Research Institute® (PCORI®) Eugene Washington PCORI Engagement Awards (#8285-BU and #EAIN-00147). The views presented here are solely the responsibility of the author(s) and do not necessarily represent the views of PCORI®, its Board of Governors or Methodology Committee. #6 © 2021 MMHRC

Engaging Mothers Through Instagram

Instagram is one of the most popular social media platforms. These tips will help you engage with mothers.

Follow a 40/40/20 content rule of thumb:

- 40% educational/informational: inform or share an important insight into a common problem.
- 40% inspirational/aspirational: describe what you hope to accomplish with your research.
- 20% direct "calls to action": actively promote your study and ask for participants.

Include very specific, relevant hashtags with your post.

- Hashtags function as a topic or heading to organize information and make finding content easier.
- Put up to 30 hashtags in the first comment on your post, not in the caption that goes with your image.

Record and post a short video about your research and why your study is important.

Share information about yourself to build trust. Trust helps mothers choose to follow you.

Always respond to comments on your posts as soon as possible. Speedy responses sustain interest in your work.

MMHRC
Maternal Mental Health Research Collaborative

Brandeis | THE HELLER SCHOOL FOR SOCIAL POLICY AND MANAGEMENT
Institute for Behavioral Health

research4moms (Facebook)
research4moms (Instagram)
research4mom (Twitter)

research4moms.com

heller.brandeis.edu/ibh/affiliates/mmhrc

linkedin.com/company/25065411

Funded through Patient-Centered Outcomes Research Institute® (PCORI®) Eugene Washington PCORI Engagement Awards (#8285-BU and #EAIN-00147). The views presented here are solely the responsibility of the author(s) and do not necessarily represent the views of PCORI®, its Board of Governors or Methodology Committee. #7 © 2021 MMHRC

Mothers' Participation Throughout the Research Lifecycle

Mothers can provide useful advice on all aspects of research projects. Consider their perspectives and how they might be involved in each of these activities.

- Prioritizing research topics.
- Increasing community relevance
- Strengthening recruitment and retention efforts.
- Framing and reviewing protocols and procedures.
- Participating in data collection and analysis.
- Interpreting findings.
- Promoting the dissemination and use of results.

MM:RC Maternal Mental Health Research Collaborative

research4moms.com

Brandeis | THE HELLER SCHOOL FOR SOCIAL POLICY AND MANAGEMENT Institute for Behavioral Health

heller.brandeis.edu/ibh/affiliates/mmhrc

research4moms

research4moms

research4mom

linkedin.com/company/25065411

Funded through Patient-Centered Outcomes Research Institute® (PCORI®) Eugene Washington PCORI Engagement Awards (#8285-BU and #EAIN-00147). The views presented here are solely the responsibility of the author(s) and do not necessarily represent the views of PCORI®, its Board of Governors or Methodology Committee. #8 © 2021 MMHRC

How Do You Know Which Health Information to Trust?

Making decisions for yourself and your family can be confusing when there are so many opinions out there about health and illness. Here are some tips for getting reliable, factual information.

Where is the information coming from? Is the source reliable, like a well-trained healthcare provider, reputable professional or advocacy organization, or well-known university?

Search for the same information from more than once source. Look for information that relates to you, your family, and your circumstances.

Information may change as researchers learn more about medical conditions and treatments. Keep asking questions.

Try these trusted sources: cdc.gov; medlineplus.gov.

MMHRC
Maternal Mental Health Research Collaborative

research4moms.com

Brandeis
THE HELLER SCHOOL FOR SOCIAL POLICY AND MANAGEMENT
Institute for Behavioral Health

heller.brandeis.edu/ibh/affiliates/mmhrc

research4moms
research4moms
research4mom
linkedin.com/company/25065411

Funded through Patient-Centered Outcomes Research Institute® (PCORI®) Eugene Washington PCORI Engagement Awards (#8285-BU and #EAIN-00147). The views presented here are solely the responsibility of the author(s) and do not necessarily represent the views of PCORI®, its Board of Governors or Methodology Committee. #9 © 2021 MMHRC

Finding Other Mothers for Peer Support

Connecting with others is an important part of staying well. Here are some tips for reaching out to feel less isolated.

Schedule regular check-ins with your friends or family by phone or text as part of your daily routine.

Join a group activity - that brings mothers together in a way that works with your schedule and transportation options, such as a mothers', spiritual, exercise, or book group

Tell someone you trust if you are concerned about using substances you depend on to cope.

Search for online support group meetings offered by your local Alcoholics Anonymous (AA), Narcotics Anonymous (NA) and other support organizations.

Create a support plan in advance that has the names and phone numbers of the people you can rely on when you are struggling.

MMHRC
Maternal Mental Health Research Collaborative

Brandeis | THE HELLER SCHOOL FOR SOCIAL POLICY AND MANAGEMENT Institute for Behavioral Health

research4moms

research4moms

research4mom

research4moms.com

heller.brandeis.edu/ibh/affiliates/mmhrc

linkedin.com/company/25065411

Funded through Patient-Centered Outcomes Research Institute® (PCORI®) Eugene Washington PCORI Engagement Awards (#8285-BU and #EAIN-00147). The views presented here are solely the responsibility of the author(s) and do not necessarily represent the views of PCORI®, its Board of Governors or Methodology Committee. #10 © 2021 MMHRC

Sharing Research Findings to Inform Mothers' Health Decision-Making

It can be hard for mothers to make good decisions for themselves and their families when so much information and so many opinions are available. Here are some tips for sharing reliable, research-based findings.

Keep it clean and simple. Use plain language in bulleted text to make materials easy to read and understand.

Make it accessible. Create materials in multiple formats (e.g., printable text, videos, photos) to convey your message through social media, email, and paper flyers.

Repeat your message multiple times. For information to be remembered, people need to see it more than once.

Present findings in different ways. Incorporate graphics, images, videos, and other media to help explain main points.

Seek non-professional avenues to share your findings. Peers and social networks are important sources of information for mothers.

MMHRC
Maternal Mental Health Research Collaborative

Brandeis | THE HELLER SCHOOL FOR SOCIAL POLICY AND MANAGEMENT Institute for Behavioral Health

research4moms
research4moms
research4mom
linkedin.com/company/25065411

research4moms.com

heller.brandeis.edu/ibh/affiliates/mmhrc

Funded through Patient-Centered Outcomes Research Institute® (PCORI®) Eugene Washington PCORI Engagement Awards (#8285-BU and #EAIN-00147). The views presented here are solely the responsibility of the author(s) and do not necessarily represent the views of PCORI®, its Board of Governors or Methodology Committee. #11 © 2021 MMHRC
